# Causal Mediation of Immune Cells and Fatty Acids in Coronary Atherosclerosis: Insights From Mendelian Randomization Analysis

**DOI:** 10.1155/humu/1253577

**Published:** 2026-05-23

**Authors:** Yong Zhang, Jia Xu, Zhihao Deng, Xiang Long, Jukun Liu, Linhui Jiang, Nanbo Liu, Dengfeng Zhang, Wenkai Zhou, Ping Zhu

**Affiliations:** ^1^ School of Medicine, South China University of Technology, Guangzhou, Guangdong, China, scut.edu.cn; ^2^ Department of Cardiovascular Surgery, Guangdong Provincial People′s Hospital, Guangdong Academy of Medical Sciences, Southern Medical University, Guangzhou, China, fimmu.com; ^3^ Guangdong Provincial Key Laboratory of South China Structural Heart Disease, Guangzhou, Guangdong, China; ^4^ Guangdong Provincial Key Laboratory of Pathogenesis, Targeted Prevention and Treatment of Heart Disease, Guangzhou Key Laboratory of Cardiac Pathogenesis and Prevention, Guangzhou, China

**Keywords:** coronary atherosclerosis, fatty acid levels, immune cells, Mendelian randomization, nutrition

## Abstract

Observational studies have suggested a link between total fatty acid levels and the development of coronary atherosclerosis. While the involvement of immune cells in the pathogenesis of this condition is well‐established, the precise causal mechanisms by which fatty acids influence immune cell function and subsequently affect coronary atherosclerosis remain unclear. To address this, we systematically assessed the causal relationships among 731 immune cell traits, circulating fatty acid levels, and coronary atherosclerosis using a two‐sample Mendelian randomization (MR) approach. Multivariable Mendelian randomization (MVMR) was further employed to investigate the potential mediating role of dendritic cells in the pathway linking fatty acids to coronary atherosclerosis. Our analysis identified significant causal associations between 30 immune cell traits and coronary atherosclerosis. Furthermore, circulating fatty acid levels were causally linked to an increased risk of coronary atherosclerosis. MVMR analysis revealed that dendritic cells, specifically the CD62L− CD86+ myeloid subset, partially mediate the causal effect of fatty acid levels on coronary atherosclerosis. This study provides genetic evidence supporting the causal roles of multiple immune cell traits and fatty acid levels in coronary atherosclerosis. Importantly, dendritic cells were identified as a key mediator in the pathway through which fatty acids influence disease risk. These findings offer new insights into the interplay between nutrition and immunity in atherosclerosis and highlight potential targets for future therapeutic and preventive strategies.

## 1. Introduction

Coronary atherosclerosis (CA) stands as a predominant contributor to cardiovascular morbidity, marked by the deposition of lipids and inflammatory cells within the arterial wall [1]. Atherosclerosis is a chronic inflammatory immunological disorder [2]. The inflammatory response is regarded as a primary driving force in the formation of atherosclerotic plaques, which can evolve into unstable plaques and even rupture. The involvement of immune cells in the pathogenesis of CA has attracted considerable scrutiny in recent years [3, 4]. The significant infiltration of various immune cells, such as monocytes/macrophages, dendritic cells (DCs), T cells, and B cells, within atherosclerotic plaques plays a crucial role in the progression of this disease [5–9]. For instance, during the progression of the disease, monocytes differentiate into macrophages and express a substantial quantity of scavenger receptors (SRs). Then, macrophages utilize the SR‐mediated endocytic pathway to eliminate modified lipoproteins, resulting in the formation of foam cells [10]. Simultaneously, macrophages release a plethora of targeted chemokines, facilitating the accumulation of monocytes/macrophages at the lesion site, which ultimately leads to plaque rupture and accelerates the progression of atherosclerosis [11].

Concurrently, the interplay between fatty acids and CA has been the subject of extensive investigation. Fatty acids, particularly polyunsaturated fatty acids (PUFAs), have been linked to both beneficial and adverse outcomes concerning cardiovascular health. The downstream metabolic products of PUFAs, such as arachidonic acid (ARA), prostaglandin (PG), thromboxane (TX), and leukotriene (LT), exert a significant influence on the onset of atherosclerosis [12–15]. Otherwise, both PUFAs themselves and the key enzyme fatty acid desaturase (FADS), which regulates their metabolism, play equally critical roles in the pathogenesis of atherosclerosis [16, 17]. Epidemiological data suggest that elevated levels of omega‐3 fatty acids (*ω*‐3 PUFA) correlate with a diminished risk of CA [18], whereas trans fatty acids may exacerbate atherosclerotic processes [19]. The underlying mechanisms governing these associations remain elusive, particularly in terms of how fatty acids modulate immune cell functionality and contribute to atherosclerosis. Emerging evidence indicates that fatty acids may influence the functional dynamics of immune cells, thereby shaping their role in atherosclerosis [20]. For instance, specific fatty acids can modify the expression of surface markers on DCs and alter their cytokine secretion, potentially affecting T‐cell activation and the broader inflammatory milieu [21].

Comprehending these interactions is essential for unraveling the intricate pathways that connect fatty acids, immune cells, and CA. However, currently, there are no studies that have systematically evaluated whether specific immune cell phenotypes mediate the causal relationship between fatty acid levels and CA. To address this critical gap in the literature, this investigation seeks to elucidate the causal relationships among immune cells, fatty acids, and CA through a Mendelian randomization (MR) framework. By comprehensively dissecting the interplay among these variables, we aspire to provide insights that may guide therapeutic strategies aimed at modulating both lipid profiles and immune responses in the prevention and management of CA.

## 2. Methods

### 2.1. Data Sources

Public access to summary statistics for each immunophenotype is available through the GWAS Catalog, with accession numbers ranging from GCST90001391 to GCST90002121. The specific GWAS Catalog IDs for the studies include ebi‐a‐GCST90092987 for research on fatty acid levels and finn‐b‐I9_CORATHER for investigations into CA.

### 2.2. Two‐Sample MR Analysis

Employing a two‐sample MR strategy, we endeavored to elucidate the causal relationships between immune cell populations and CA, as well as the influence of fatty acid levels on both CA and immune cell function. The significance level for instrumental variables (IVs) for each immunophenotype and fatty acid levels was set at 1 × 10^−5^. To ensure independence, these single‐nucleotide polymorphisms (SNPs) were pruned for linkage disequilibrium (LD) with a threshold of *r* ^2^ < 0.1. Genetic correlations, encompassing beta coefficients and their corresponding standard deviations, for these SNPs associated with immune cell profiles, serum fatty acid concentrations, and CA were extracted from their respective genome‐wide association study (GWAS) consortia. The estimation of causal influences was executed utilizing the inverse‐variance weighted (IVW) approach, which amalgamates effect size estimates derived from multiple SNPs, factoring in their relative precision. To ascertain the robustness of our findings, a suite of sensitivity analyses was conducted, encompassing MR‐Egger regression and the weighted median method. The significance level for IVs for each immunophenotype and fatty acid levels was set at *p* < 1 × 10^−5^. This relatively relaxed threshold, as opposed to the conventional genome‐wide significance threshold of *p* < 5 × 10^−8^, was selected because specific immune cell traits often lack sufficient SNPs reaching the stricter significance level due to limited sample sizes or the highly polygenic nature of these phenotypes. Employing this relaxed threshold is a well‐established approach in MR studies involving complex omics data, as it ensures the inclusion of an adequate number of independent IVs. A sufficient number of IVs is essential for performing robust causal estimations and conducting comprehensive sensitivity analyses, such as the MR‐Egger regression and the weighted median method, which require multiple instruments to detect and adjust for potential horizontal pleiotropy.

### 2.3. Multivariable Mendelian Randomization (MVMR) Analysis

To elucidate the potential mediating role of DCs in the nexus between fatty acid levels and CA, we executed an MVMR analysis. This analytical framework permits the quantification of both direct and indirect effects by integrating multiple exposures within the analytical model. For the selection of IVs in MVMR, we identified SNPs that exhibited associations with both fatty acid levels and DC function, employing these as IVs for our analysis. To ascertain the independence of these SNPs, they underwent LD pruning, applying a stringent threshold of *r* ^2^ < 0.1. Within the MVMR analysis, we delineated the direct impact of fatty acid levels on CA, with adjustments made for the confounding influence of DCs. Concurrently, we quantified the indirect effects mediated through the intermediary role of DCs.

### 2.4. Statistical Analysis

All statistical analyses were conducted utilizing R software (Version 4.3.3) in conjunction with the TwoSampleMR package (Version 0.5.1). This methodology enables the dissection of the intricate interplay among fatty acid levels, DCs, and CA, thereby affording a more nuanced comprehension of the mechanisms through which these variables exert their reciprocal influences.

## 3. Results

**Figure 1:**
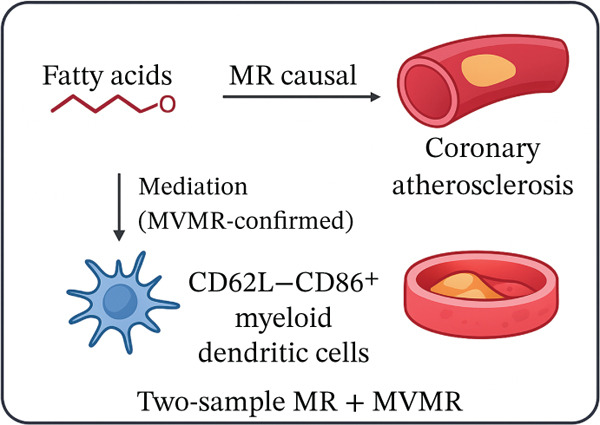
Schematic overview of the study design.

To elucidate the causal interplay between immunophenotypes, fatty acids, and CA, we employed a multistep MR framework. Our investigation first established the causal role of immune cells, then examined the influence of fatty acids, and finally integrated these findings to identify a key mediating pathway. The overall design and primary conclusions of this study are visually synthesized in Figure 1, which provides a conceptual overview of our analytical approach and central findings.

### 3.1. Causal Effects of Immune Cell Traits on Coronary Atherosclerosis

**Figure 2:**
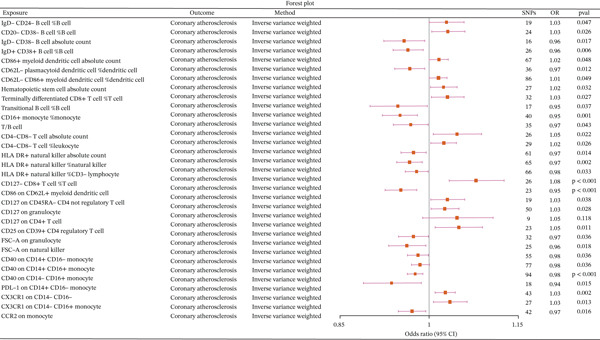
Forest plot illustrating the causal relationships between 32 out of 731 immune cell characteristics and coronary atherosclerosis, as analyzed using the inverse‐variance weighted (IVW) method.

We assessed the causal relationships between 731 immune cell traits and CA using the IVW method. Our results identified 32 immune cell traits with a causal relationship with CA (Figure 2 and Supporting Information 1: Table S1). Heterogeneity (Supporting Information 1: Table S2) and horizontal pleiotropy (Supporting Information 1: Table S3) tests further supported our findings, with *p* values greater than 0.05 indicating no evidence of heterogeneity or pleiotropy. To rigorously distinguish between unidirectional and bidirectional causality, we performed a reverse MR analysis on these 32 identified traits. The results confirmed that 30 of these immune cell phenotypes exhibit a purely unidirectional causal effect on CA, as no reverse causal relationship was observed (*p* value > 0.05). However, two specific traits—CD127 on CD45RA− CD4 not regulatory T cells (Tregs) and CD62L− plasmacytoid DC—maintained significant causal links in the reverse direction, indicating a bidirectional relationship. This clarification confirms that, while all 32 traits are causally linked to CA, 30 act solely as drivers, whereas 2 are involved in a pathological feedback loop with the disease.

Our findings indicate that several monocyte phenotypes serve as protective factors for CA, including CD40 on CD14+CD16− monocytes and CCR2 on monocytes. HLA‐DR+ natural killer cells were also identified as protective factors. Conversely, multiple T‐cell phenotypes were associated with an increased risk of CA, such as CD127− CD8+ T cells %T cell, CD4−CD8− T‐cell absolute count, and terminally differentiated CD8+ T cells %T cell. Additionally, CD62L− CD86+ myeloid DCs %DC were found to be a risk factor for CA. Inflammatory and immunological responses are pivotal in the pathogenesis of atherosclerosis and its associated vascular complications [22, 23]. Immune reactions triggered by apolipoproteins and bioactive components residing within the lipid matrix of atheromatous deposits have been implicated in the inflammatory cascade [24]. Such antigens are posited to draw the attention of antigen‐presenting cells (APCs), subsequently leading to the activation of T lymphocytes within the atherosclerotic lesion. DCs, the most formidable APCs endowed with the singular capacity to initiate a primary immune response to specific antigens [25], have been recently identified to be significantly augmented in atherosclerotic plaques [26, 27] and are actively implicated in the pathophysiological processes of atherosclerosis.

### 3.2. Causal Relationship Between Fatty Acid Levels and CA

**Figure 3:**
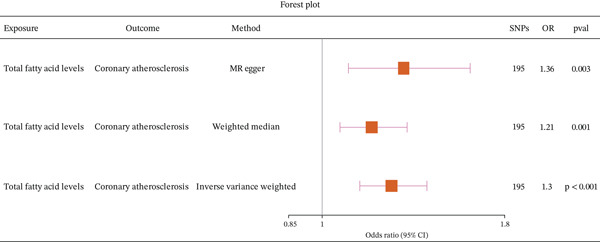
Forest plot showing the causal relationship between fatty acid levels and coronary atherosclerosis using the inverse‐variance weighted (IVW) method.

**Figure 4:**
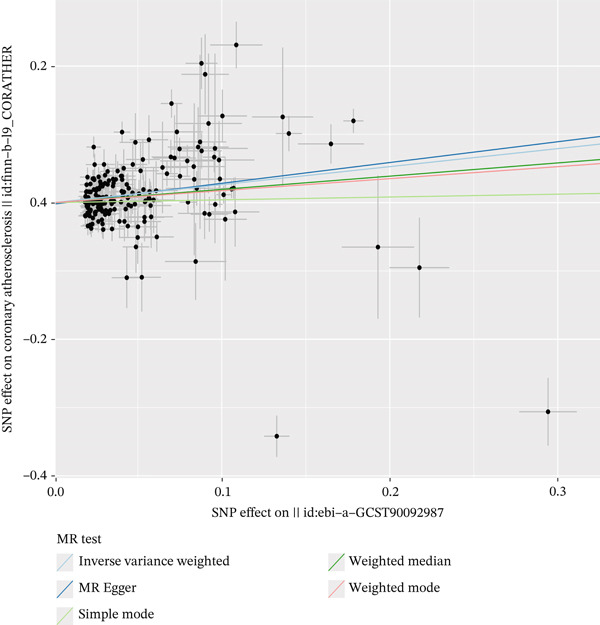
Scatter plots showing the robustness of the causal relationship between fatty acid levels and coronary atherosclerosis across multiple methods.

A plethora of research has underscored the substantial impact of fatty acid consumption on the etiology of CA. To further investigate this relationship, we conducted an MR analysis. Our results indicate that fatty acid levels have a causal relationship with CA, acting as a risk factor (Figure 3 and Supporting Information 1: Table S5). Reverse MR (Supporting Information 1: Table S6) and horizontal pleiotropy (Supporting Information 1: Table S7) assessment confirmed our findings, with scatter plots (Figure 4) and leave‐one‐out (LOO) analyses (Supporting Information 2: Figure S1) further supporting the robustness of our results.

### 3.3. Causal Relationship Between Fatty Acid Levels and Immune Cell Traits

**Figure 5:**
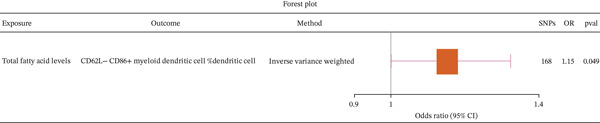
Forest plot showing the causal relationship between fatty acid levels and immune cell traits using the inverse‐variance weighted (IVW) method.

To further explore whether immune cell traits mediate the causal relationship between fatty acid levels and CA, we selected 30 immune cell traits with established causal links to CA as outcomes, with fatty acid levels as the exposure (Supporting Information 1: Table S8). Two‐sample MR analysis revealed that fatty acid levels causally contribute to an increased risk of CD62L− CD86+ myeloid DCs %DC (Figure 5). The results were validated through reverse MR (Supporting Information 1: Table S9), heterogeneity testing (Supporting Information 1: Table S10), and horizontal pleiotropy assessment (Supporting Information 1: Table S11).

### 3.4. CD62L− CD86+ Myeloid DCs Mediate the Causal Relationship Between Fatty Acid Levels and CA

**Figure 6:**
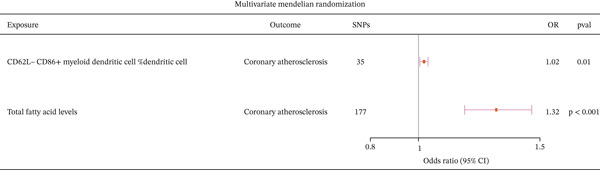
Multivariable Mendelian randomization analysis of the causal relationship between fatty acids and immune cells as exposures and coronary atherosclerosis.

To further investigate whether CD62L− CD86+ myeloid DCs mediate the causal relationship between fatty acid levels and CA, we conducted an MVMR analysis with CD62L− CD86+ myeloid DCs and fatty acid levels as exposures and CA as the outcome. The results indicated that both exposures still have a causal relationship with CA (Figure 6 and Supporting Information 1: Table S12). These findings suggest that CD62L− CD86+ myeloid DCs mediate the causal relationship between fatty acid levels and CA.

## 4. Discussion

Our study investigates the complex causal relationships among immune cell characteristics, fatty acid levels, and CA, offering novel insights into how these factors interact to shape cardiovascular health. We identified several monocyte phenotypes, such as CD40 on CD14+CD16− monocytes and CCR2 on monocytes, that exhibit protective effects against CA. Conversely, another study revealed that the proliferation of CD16+ monocytes significantly heightened the risk of coronary artery disease, thereby reinforcing our findings that CD16− monocytes attenuate the progression of atherosclerosis [28]. Additionally, research indicates that the contributions of various monocyte subsets—classical CD14++CD16−, intermediate CD14++CD16+, and nonclassical CD14+CD16+—to atherosclerosis development may vary with age [29]. Monocytes are not a homogeneous cell population; their distinct subsets may play divergent roles in inflammatory responses. Our investigation specifically highlights the protective role of CD14+CD16− monocytes within inflammatory contexts, while other subsets, such as CD14+CD16+ monocytes, may facilitate the progression of atherosclerosis. Consequently, future research should further delineate the functional characteristics of these subsets and their specific roles in atherosclerosis. Furthermore, HLA‐DR+ natural killer cells were also identified as protective factors. Studies suggest that the atherogenic effects of LDL cholesterol may be mitigated by HDL cholesterol through the anti‐inflammatory cytokine IL‐10 and HLA‐DR [30]. These cells are essential for immune surveillance and have been implicated in the regulation of inflammation and cellular stress, which may help to mitigate the progression of CA.

T cells engage in clonal expansion and interact with other cells within the plaque, playing a pivotal role in the initiation, progression, rupture, and erosion of atherosclerotic plaques, thereby influencing the advancement of atherosclerosis either by accelerating or by decelerating its course [31]. In the presence of APCs, T cells differentiate into specific functional subsets, including helper T cells (such as Th1, Th2, Th9, and Th17) and Tregs, driven by variations in the expression of transcription factors [32]. These subsets modulate the immune response in atherosclerosis through the secretion of a diverse array of proinflammatory and anti‐inflammatory cytokines. Furthermore, based on distinct surface differentiation antigens, T cells can be classified into two major subsets: CD4+ and CD8+ [33, 34]. The CD4+ T‐cell subset exhibits distinct transcriptional programs and cytokine secretion profiles. Influenced by costimulatory signals provided by APCs and the diverse cytokine milieu within the microenvironment, CD4+ T cells can differentiate into various T‐cell subsets. These subsets exert either proinflammatory or anti‐inflammatory effects by secreting cytokines and chemokines that activate and recruit target cells [35, 36]. Consequently, CD4+ T cells exhibit a dual role in atherosclerosis, either accelerating or inhibiting its progression, contingent upon the specific cytokines they secrete. CD8+ T cells are integral components of both innate and adaptive immune defense mechanisms, regulating the immune response by exerting cytotoxic functions against exogenous threats and endogenous risks. Upon the binding of the T‐cell receptor (TCR) on CD8+ T cells to antigenic peptides presented by major histocompatibility complex (MHC) Class I molecules on APCs, CD8+ T cells become activated. This activation leads to clonal proliferation and differentiation into effector T cells, which subsequently modulate atherosclerosis [37]. Current research indicates that CD8+ T cells play both promoting and inhibitory roles in atherosclerosis, with their effects potentially varying depending on the distinct stages of atherosclerotic lesion development. On one hand, CD8+ T cells can promote the recruitment of monocytes and increase plaque vulnerability, indicating their proatherogenic role [38, 39]. On the other hand, CD8+ T cells can initiate cytolytic responses against proatherogenic DCs and follicular helper T cells, thereby restricting the progression of atherosclerosis [40, 41].

Our MR analysis has elucidated a causal relationship between fatty acid levels and CA, demonstrating that elevated levels of fatty acids are linked to an increased risk of developing this condition. This finding is consistent with existing literature that suggests dietary fats, particularly saturated and trans fats, play a significant role in the pathogenesis of atherosclerosis through various mechanisms, including inflammation and endothelial dysfunction [19, 42]. Furthermore, the application of reverse MR, alongside heterogeneity testing and assessments of horizontal pleiotropy, served to validate these results, thereby reinforcing the robustness and reliability of our findings. Further analysis demonstrated that fatty acid levels have a causal impact on the proportion of CD62L− CD86+ myeloid DCs within the total DC population. This relationship implies that fatty acids may play a significant role in influencing immune cell profiles, potentially through mechanisms such as the modulation of inflammatory pathways or direct effects on immune cell functionality. The identification of this association highlights the importance of fatty acids in shaping the immune landscape, which could subsequently influence the development of atherosclerosis. Understanding this link may provide valuable insights into the interplay between dietary components and immune responses, ultimately contributing to our knowledge of cardiovascular health and disease progression [43].

Additionally, our findings identified the percentage of CD62L− CD86+ myeloid DCs within the DC population as a significant risk factor. These DCs are pivotal in antigen presentation and T‐cell activation, potentially perpetuating inflammation and immune responses that further exacerbate the pathogenesis of atherosclerosis [44]. MVMR analysis revealed that CD62L− CD86+ myeloid DCs serve as significant mediators in the causal relationship between fatty acid levels and CA. This finding indicates that fatty acids may exert their influence on CA through their impact on specific DC subsets, which play a crucial role in modulating immune responses and inflammation. CD62L− CD86+ myeloid DCs are particularly recognized for their involvement in antigen presentation and the activation of immune responses, and they may drive inflammatory processes that contribute to the acceleration of atherosclerosis [45]. Understanding this mediating role enhances our comprehension of how dietary fatty acids can shape immune cell dynamics, ultimately influencing the pathogenesis of cardiovascular diseases.

Notably, our reverse MR analysis revealed bidirectional causal relationships for two specific immune phenotypes: CD127 expression on CD45RA− CD4 non‐Tregs (memory CD4+ T cells) and the percentage of CD62L− plasmacytoid DCs. The biological plausibility of this bidirectional link likely stems from a complex, pathological feedback loop between systemic immunity and the atherosclerotic microenvironment. On one hand, these immune subsets contribute to the pathogenesis of CA. On the other hand, the established atherosclerotic state—characterized by chronic low‐grade inflammation, continuous antigen presentation, and the release of damage‐associated molecular patterns (DAMPs) from necrotic core formation—can systemically shape the immune profile. For instance, this chronic inflammatory milieu may alter the homeostatic proliferation and survival of memory CD4+ T cells by dynamically modulating CD127 (the interleukin‐7 receptor) expression. Similarly, ongoing vascular tissue damage can continuously activate circulating plasmacytoid DCs, prompting the shedding of CD62L (L‐selectin)—a hallmark of immune activation and tissue migration. Consequently, these specific immune phenotypes not only drive atherosclerosis but also are concurrently and continuously reshaped by the disease itself.

Our study presents several limitations that warrant consideration. First, a potential limitation inherent to our two‐sample MR design is the possibility of sample overlap between the exposure and outcome GWAS datasets. Although we purposefully extracted data from distinct consortia—utilizing the broader GWAS Catalog for immune cell traits and fatty acids and the FinnGen consortium for CA—to maximize sample independence, the pervasive sharing of control cohorts across large‐scale genetic studies makes completely ruling out participant overlap challenging. Sample overlap can potentially bias causal estimates toward observational associations, which is particularly problematic when relying on weak instruments. We mitigated this risk by strictly retaining IVs with an F‐statistic greater than 10 to ensure robust instrument strength; nevertheless, future research utilizing strictly independent cohorts or individual‐level data is warranted to definitively validate these causal pathways. Second, our analysis treated “circulating fatty acid levels” as a single, aggregated exposure entity. Epidemiological and biological data strongly suggest that different classes of fatty acids exhibit diverse and often opposing effects on cardiovascular health; for instance, elevated levels of omega‐3 fatty acids correlate with a diminished risk of CA, whereas trans fatty acids can exacerbate atherosclerotic processes. Because we relied on a generalized GWAS dataset for overall fatty acid levels, we were unable to stratify these causal effects by specific fatty acid subtypes, meaning the observed relationships represent a net or generalized impact. Similarly, our study was unable to distinguish between the precise biological sources of these circulating fatty acids. The genetic instruments proxy overall levels, which inherently represent a complex interplay between exogenous dietary intake and endogenous metabolic processes, such as the regulatory actions of FADS enzymes. Consequently, we cannot definitively disentangle the effects of diet‐derived fatty acids from those endogenously synthesized. This distinction is critical for translating our findings into actionable dietary recommendations. While our results robustly link elevated overall fatty acid levels to an increased risk of CA via immune mediation, they must be interpreted cautiously within a nutritional context. Future MR studies utilizing more granular, high‐resolution GWAS data, coupled with advanced metabolomics, are urgently needed to systematically evaluate the specific pathways of individual fatty acid classes and their distinct sources. Finally, while our analysis identified specific immune cell traits that causally influence CA, it is equally important to acknowledge that the vast majority of the 731 evaluated immune cell phenotypes did not exhibit a significant causal relationship with the disease. These null findings offer valuable insights. On one hand, they may reflect a true absence of a systemic causal effect, suggesting that many immune cell subsets function primarily in a reactive, rather than a driving, capacity during disease progression. On the other hand, these negative results could be attributed to statistical power limitations, as GWAS for certain rare or highly specific immune subphenotypes may currently lack the sample size required to detect modest causal effects. Furthermore, the dynamic nature of immune cells means their functional roles can be highly context‐dependent; some may exert localized, transient effects within the atherosclerotic plaque microenvironment that are not adequately captured by systemic circulating profiles. Future research utilizing larger cohorts and tissue‐specific multiomics approaches will be essential to definitively differentiate true null effects from those masked by current analytical limitations.

Our study provides significant insights into the mechanisms linking fatty acid levels, immune cell phenotypes, and CA. By identifying specific immune cell traits associated with atherosclerosis and elucidating the mediating role of DCs, our findings highlight potential therapeutic targets for managing and preventing CA. These results suggest that strategies aimed at modifying fatty acid intake and targeting specific immune cell subsets could offer new avenues for intervention. The integration of dietary factors with immune modulation presents a novel approach to understanding and addressing the pathophysiology of CA, potentially leading to more effective preventive and therapeutic strategies.

## Author Contributions

Ping Zhu, Wenkai Zhou, and Nanbo Liu conceived and designed the study. Dengfeng Zhang, Yong Zhang, Jia Xu, Xiang Long, Linhui Jiang, and Jukun Liu performed the research. Ping Zhu, Dengfeng Zhang, Jia Xu, Yong Zhang, and Linhui Jiang analyzed the data. Dengfeng Zhang, Yong Zhang, Zhihao Deng, and Xiang Long wrote the manuscript. Dengfeng Zhang, Wenkai Zhou, and Ping Zhu are co‐corresponding authors.

## Funding

This work was supported by the Science and Technology Planning Project of Guangdong Province (No. 2022B1212010010), the National Outstanding Youth Science Fund Project of National Natural Science Foundation of China (10.13039/100014717, No. 32400765), the 2024 Stability Support for Innovative Capacity Building of Guangdong Provincial Scientific Research Institutions (No. KD022024023), and the National Natural Science Foundation of China (10.13039/501100001809, No. 82370353).

## Ethics Statement

Ethical approval was not necessary for this study.

## Conflicts of Interest

The authors declare no conflicts of interest.

## Supporting Information

Additional supporting information can be found online in the Supporting Information section.

## Supporting information


**Supporting Information 1** Table S1: Analysis of the causal relationship between 731 immune cell characteristics and coronary atherosclerosis using the inverse‐variance weighted (IVW) method. Table S2: The heterogeneity test results for the causal relationships between 32 immune cell characteristics and coronary atherosclerosis. Table S3: The horizontal pleiotropy test results for the causal relationships between 32 immune cell characteristics and coronary atherosclerosis. Table S4: Reverse Mendelian randomization analysis of the causal relationship between 32 immune cell traits and coronary atherosclerosis. Table S5: Mendelian randomization analysis of the causal relationship between fatty acid levels and coronary atherosclerosis. Table S6: Reverse Mendelian randomization analysis of the causal relationship between fatty acid levels and coronary atherosclerosis. Table S7: Horizontal pleiotropy test for fatty acid levels and coronary atherosclerosis. Table S8: Mendelian randomization analysis of the causal relationship between fatty acid levels and immune cell traits. Table S9: Reverse Mendelian randomization analysis of the causal relationship between fatty acid levels and immune cell traits. Table S10: Heterogeneity test for fatty acid levels and immune cell traits. Table S11: Horizontal pleiotropy test for fatty acid levels and immune cell traits. Table S12: Multivariable Mendelian randomization analysis of the causal relationship between fatty acids, immune cells, and coronary atherosclerosis.

**Supporting Information 2** Figure S1: Leave‐one‐out (LOO) analyses.

## Data Availability

The summary‐level datasets underpinning this study′s analysis are openly accessible. Specifically, the data were retrieved via the IEU OpenGWAS project repository, which can be accessed at https://gwas.mrcieu.ac.uk/. This platform aggregates and standardizes GWAS summary statistics from primary sources, including the EBI GWAS Catalog and the FinnGen consortium. Detailed accession numbers for all specific exposures and outcomes are provided in the Methods section and the Supporting Information.
