# Supplementary material for: Causal Mediation of Immune Cells and Fatty Acids in Coronary Atherosclerosis: Insights From Mendelian Randomization Analysis
Source: Hum Mutat. 2026 May 23;2026:1253577. doi: 10.1155/humu/1253577 (PMC13197653; doi:10.1155/humu/1253577)
Supplement: Supplementary file 1 — Supporting Information 1 Table S1: Analysis of the causal relationship between 731 immune cell characteristics and coronary atherosclerosis using the inverse‐variance weighted (IVW) method. Table S2: The heterogeneity test results for the causal relationships between 32 immune cell characteristics and coronary atherosclerosis. Table S3: The horizontal pleiotropy test results for the causal relationships between 32 immune cell characteristics and coronary atherosclerosis. Table S4: Reverse Mendelian randomization analysis of the causal relationship between 32 immune cell traits and coronary atherosclerosis. Table S5: Mendelian randomization analysis of the causal relationship between fatty acid levels and coronary atherosclerosis. Table S6: Reverse Mendelian randomization analysis of the causal relationship between fatty acid levels and coronary atherosclerosis. Table S7: Horizontal pleiotropy test for fatty acid levels and coronary atherosclerosis. Table S8: Mendelian randomization analysis of the causal relationship between fatty acid levels and immune cell traits. Table S9: Reverse Mendelian randomization analysis of the causal relationship between fatty acid levels and immune cell traits. Table S10: Heterogeneity test for fatty acid levels and immune cell traits. Table S11: Horizontal pleiotropy test for fatty acid levels and immune cell traits. Table S12: Multivariable Mendelian randomization analysis of the causal relationship between fatty acids, immune cells, and coronary atherosclerosis. [file HUMU-2026-1253577-s001.docx]

| **Table S1:** Analysis of the causal relationship between 731 immune cell characteristics and coronary atherosclerosis using the Inverse Variance Weighting (IVW) method. | | | | | | | | | | | | | | |
| --- | --- | --- | --- | --- | --- | --- | --- | --- | --- | --- | --- | --- | --- | --- |
| id.exposure | exposure | id.outcome | outcome | exposure | method | nsnp | b | se | pval | lo_ci | up_ci | or | or_lci95 | or_uci95 |
| ebi-a-GCST90001413 | IgD- CD24- B cell %B cell | finn-b-I9_CORATHER | Coronary atherosclerosis \|\| id:finn-b-I9_CORATHER | \|\| id:ebi-a-GCST90001413 | Inverse variance weighted | 19 | 0.028365 | 0.014293 | 0.047197 | 0.000351 | 0.05638 | 1.028771 | 1.000351 | 1.058 |
| ebi-a-GCST90001422 | CD20- CD38- B cell %B cell | finn-b-I9_CORATHER | Coronary atherosclerosis \|\| id:finn-b-I9_CORATHER | \|\| id:ebi-a-GCST90001422 | Inverse variance weighted | 24 | 0.030582 | 0.013701 | 0.025613 | 0.003727 | 0.057436 | 1.031054 | 1.003734 | 1.059118 |
| ebi-a-GCST90001446 | IgD- CD38- B cell Absolute Count | finn-b-I9_CORATHER | Coronary atherosclerosis \|\| id:finn-b-I9_CORATHER | \|\| id:ebi-a-GCST90001446 | Inverse variance weighted | 16 | -0.04345 | 0.018125 | 0.016509 | -0.07898 | -0.00793 | 0.957477 | 0.92406 | 0.992103 |
| ebi-a-GCST90001447 | IgD+ CD38+ B cell %B cell | finn-b-I9_CORATHER | Coronary atherosclerosis \|\| id:finn-b-I9_CORATHER | \|\| id:ebi-a-GCST90001447 | Inverse variance weighted | 26 | -0.03694 | 0.013351 | 0.005658 | -0.06311 | -0.01077 | 0.963732 | 0.938841 | 0.989284 |
| ebi-a-GCST90001464 | CD86+ myeloid Dendritic Cell Absolute Count | finn-b-I9_CORATHER | Coronary atherosclerosis \|\| id:finn-b-I9_CORATHER | \|\| id:ebi-a-GCST90001464 | Inverse variance weighted | 67 | 0.01559 | 0.0079 | 0.048447 | 0.000106 | 0.031075 | 1.015713 | 1.000106 | 1.031563 |
| ebi-a-GCST90001471 | CD62L- plasmacytoid Dendritic Cell %Dendritic Cell | finn-b-I9_CORATHER | Coronary atherosclerosis \|\| id:finn-b-I9_CORATHER | \|\| id:ebi-a-GCST90001471 | Inverse variance weighted | 36 | -0.03364 | 0.013384 | 0.01196 | -0.05987 | -0.00741 | 0.966921 | 0.941886 | 0.992622 |
| ebi-a-GCST90001473 | CD62L- CD86+ myeloid Dendritic Cell %Dendritic Cell | finn-b-I9_CORATHER | Coronary atherosclerosis \|\| id:finn-b-I9_CORATHER | \|\| id:ebi-a-GCST90001473 | Inverse variance weighted | 86 | 0.014834 | 0.007546 | 0.049304 | 4.49E-05 | 0.029624 | 1.014945 | 1.000045 | 1.030067 |
| ebi-a-GCST90001514 | Hematopoietic Stem Cell Absolute Count | finn-b-I9_CORATHER | Coronary atherosclerosis \|\| id:finn-b-I9_CORATHER | \|\| id:ebi-a-GCST90001514 | Inverse variance weighted | 27 | 0.023796 | 0.011091 | 0.031915 | 0.002057 | 0.045535 | 1.024081 | 1.002059 | 1.046588 |
| ebi-a-GCST90001559 | Terminally Differentiated CD8+ T cell %T cell | finn-b-I9_CORATHER | Coronary atherosclerosis \|\| id:finn-b-I9_CORATHER | \|\| id:ebi-a-GCST90001559 | Inverse variance weighted | 32 | 0.030063 | 0.013584 | 0.026885 | 0.003439 | 0.056687 | 1.03052 | 1.003445 | 1.058325 |
| ebi-a-GCST90001576 | Transitional B cell %B cell | finn-b-I9_CORATHER | Coronary atherosclerosis \|\| id:finn-b-I9_CORATHER | \|\| id:ebi-a-GCST90001576 | Inverse variance weighted | 17 | -0.05431 | 0.025981 | 0.036574 | -0.10524 | -0.00339 | 0.947135 | 0.900112 | 0.996615 |
| ebi-a-GCST90001587 | CD16+ monocyte %monocyte | finn-b-I9_CORATHER | Coronary atherosclerosis \|\| id:finn-b-I9_CORATHER | \|\| id:ebi-a-GCST90001587 | Inverse variance weighted | 40 | -0.05067 | 0.015631 | 0.001189 | -0.0813 | -0.02003 | 0.950595 | 0.921913 | 0.980169 |
| ebi-a-GCST90001588 | T/B cell | finn-b-I9_CORATHER | Coronary atherosclerosis \|\| id:finn-b-I9_CORATHER | \|\| id:ebi-a-GCST90001588 | Inverse variance weighted | 35 | -0.03179 | 0.015683 | 0.042688 | -0.06252 | -0.00105 | 0.968715 | 0.939391 | 0.998954 |
| ebi-a-GCST90001598 | CD4-CD8- T cell Absolute Count | finn-b-I9_CORATHER | Coronary atherosclerosis \|\| id:finn-b-I9_CORATHER | \|\| id:ebi-a-GCST90001598 | Inverse variance weighted | 26 | 0.045364 | 0.019737 | 0.021539 | 0.006679 | 0.084048 | 1.046408 | 1.006701 | 1.087682 |
| ebi-a-GCST90001613 | CD4-CD8- T cell %leukocyte | finn-b-I9_CORATHER | Coronary atherosclerosis \|\| id:finn-b-I9_CORATHER | \|\| id:ebi-a-GCST90001613 | Inverse variance weighted | 29 | 0.023565 | 0.010578 | 0.025897 | 0.002832 | 0.044298 | 1.023845 | 1.002836 | 1.045294 |
| ebi-a-GCST90001648 | HLA DR+ Natural Killer Absolute Count | finn-b-I9_CORATHER | Coronary atherosclerosis \|\| id:finn-b-I9_CORATHER | \|\| id:ebi-a-GCST90001648 | Inverse variance weighted | 61 | -0.02737 | 0.011096 | 0.013645 | -0.04912 | -0.00562 | 0.973003 | 0.95207 | 0.994396 |
| ebi-a-GCST90001649 | HLA DR+ Natural Killer %Natural Killer | finn-b-I9_CORATHER | Coronary atherosclerosis \|\| id:finn-b-I9_CORATHER | \|\| id:ebi-a-GCST90001649 | Inverse variance weighted | 65 | -0.03423 | 0.011081 | 0.00201 | -0.05595 | -0.01251 | 0.966352 | 0.945589 | 0.98757 |
| ebi-a-GCST90001650 | HLA DR+ Natural Killer %CD3- lymphocyte | finn-b-I9_CORATHER | Coronary atherosclerosis \|\| id:finn-b-I9_CORATHER | \|\| id:ebi-a-GCST90001650 | Inverse variance weighted | 66 | -0.0219 | 0.010271 | 0.032974 | -0.04203 | -0.00177 | 0.978336 | 0.958838 | 0.998231 |
| ebi-a-GCST90001682 | CD127- CD8+ T cell %T cell | finn-b-I9_CORATHER | Coronary atherosclerosis \|\| id:finn-b-I9_CORATHER | \|\| id:ebi-a-GCST90001682 | Inverse variance weighted | 26 | 0.075627 | 0.021914 | 0.000558 | 0.032675 | 0.118579 | 1.07856 | 1.033214 | 1.125896 |
| ebi-a-GCST90001904 | CD86 on CD62L+ myeloid Dendritic Cell | finn-b-I9_CORATHER | Coronary atherosclerosis \|\| id:finn-b-I9_CORATHER | \|\| id:ebi-a-GCST90001904 | Inverse variance weighted | 23 | -0.04898 | 0.014063 | 0.000496 | -0.07654 | -0.02141 | 0.952203 | 0.926316 | 0.978814 |
| ebi-a-GCST90001923 | CD127 on CD45RA- CD4 not regulatory T cell | finn-b-I9_CORATHER | Coronary atherosclerosis \|\| id:finn-b-I9_CORATHER | \|\| id:ebi-a-GCST90001923 | Inverse variance weighted | 19 | 0.029538 | 0.014219 | 0.037775 | 0.001668 | 0.057407 | 1.029978 | 1.001669 | 1.059087 |
| ebi-a-GCST90001926 | CD127 on granulocyte | finn-b-I9_CORATHER | Coronary atherosclerosis \|\| id:finn-b-I9_CORATHER | \|\| id:ebi-a-GCST90001926 | Inverse variance weighted | 50 | 0.025765 | 0.01169 | 0.02752 | 0.002853 | 0.048677 | 1.0261 | 1.002857 | 1.049881 |
| ebi-a-GCST90001931 | CD127 on CD4+ T cell | finn-b-I9_CORATHER | Coronary atherosclerosis \|\| id:finn-b-I9_CORATHER | \|\| id:ebi-a-GCST90001931 | Inverse variance weighted | 9 | 0.045015 | 0.028803 | 0.118089 | -0.01144 | 0.10147 | 1.046044 | 0.988626 | 1.106796 |
| ebi-a-GCST90001935 | CD25 on CD39+ CD4 regulatory T cell | finn-b-I9_CORATHER | Coronary atherosclerosis \|\| id:finn-b-I9_CORATHER | \|\| id:ebi-a-GCST90001935 | Inverse variance weighted | 23 | 0.048181 | 0.019066 | 0.0115 | 0.010813 | 0.085549 | 1.049361 | 1.010871 | 1.089315 |
| ebi-a-GCST90001966 | FSC-A on granulocyte | finn-b-I9_CORATHER | Coronary atherosclerosis \|\| id:finn-b-I9_CORATHER | \|\| id:ebi-a-GCST90001966 | Inverse variance weighted | 32 | -0.0273 | 0.013052 | 0.036496 | -0.05288 | -0.00171 | 0.973073 | 0.948496 | 0.998287 |
| ebi-a-GCST90001969 | FSC-A on Natural Killer | finn-b-I9_CORATHER | Coronary atherosclerosis \|\| id:finn-b-I9_CORATHER | \|\| id:ebi-a-GCST90001969 | Inverse variance weighted | 25 | -0.0387 | 0.016371 | 0.018084 | -0.07079 | -0.00661 | 0.96204 | 0.931661 | 0.99341 |
| ebi-a-GCST90001980 | CD40 on CD14+ CD16- monocyte | finn-b-I9_CORATHER | Coronary atherosclerosis \|\| id:finn-b-I9_CORATHER | \|\| id:ebi-a-GCST90001980 | Inverse variance weighted | 55 | -0.01943 | 0.009277 | 0.036269 | -0.03761 | -0.00124 | 0.980762 | 0.96309 | 0.998759 |
| ebi-a-GCST90001981 | CD40 on CD14+ CD16+ monocyte | finn-b-I9_CORATHER | Coronary atherosclerosis \|\| id:finn-b-I9_CORATHER | \|\| id:ebi-a-GCST90001981 | Inverse variance weighted | 77 | -0.01567 | 0.007456 | 0.035543 | -0.03029 | -0.00106 | 0.984448 | 0.970166 | 0.998941 |
| ebi-a-GCST90001989 | CD40 on CD14- CD16+ monocyte | finn-b-I9_CORATHER | Coronary atherosclerosis \|\| id:finn-b-I9_CORATHER | \|\| id:ebi-a-GCST90001989 | Inverse variance weighted | 94 | -0.02431 | 0.006874 | 0.000405 | -0.03778 | -0.01084 | 0.975982 | 0.96292 | 0.98922 |
| ebi-a-GCST90001993 | PDL-1 on CD14+ CD16- monocyte | finn-b-I9_CORATHER | Coronary atherosclerosis \|\| id:finn-b-I9_CORATHER | \|\| id:ebi-a-GCST90001993 | Inverse variance weighted | 18 | -0.06498 | 0.026605 | 0.014594 | -0.11712 | -0.01283 | 0.937088 | 0.889475 | 0.98725 |
| ebi-a-GCST90001994 | CX3CR1 on CD14- CD16- | finn-b-I9_CORATHER | Coronary atherosclerosis \|\| id:finn-b-I9_CORATHER | \|\| id:ebi-a-GCST90001994 | Inverse variance weighted | 43 | 0.026123 | 0.008373 | 0.00181 | 0.009712 | 0.042535 | 1.026467 | 1.009759 | 1.043452 |
| ebi-a-GCST90002012 | CX3CR1 on CD14- CD16+ monocyte | finn-b-I9_CORATHER | Coronary atherosclerosis \|\| id:finn-b-I9_CORATHER | \|\| id:ebi-a-GCST90002012 | Inverse variance weighted | 27 | 0.034038 | 0.013758 | 0.01336 | 0.007072 | 0.061004 | 1.034624 | 1.007097 | 1.062903 |
| ebi-a-GCST90002017 | CCR2 on monocyte | finn-b-I9_CORATHER | Coronary atherosclerosis \|\| id:finn-b-I9_CORATHER | \|\| id:ebi-a-GCST90002017 | Inverse variance weighted | 42 | -0.02965 | 0.012253 | 0.015536 | -0.05366 | -0.00563 | 0.970788 | 0.947753 | 0.994384 |

**Table S2:** The heterogeneity test results for the causal relationships between 32 immune cell characteristics and coronary atherosclerosis.

|  | id.exposure | id.outcome | outcome | exposure | method | Q | Q_df | Q_pval |
| --- | --- | --- | --- | --- | --- | --- | --- | --- |
| 1 | ebi-a-GCST90001413 | finn-b-I9_CORATHER | Coronary atherosclerosis \|\| id:finn-b-I9_CORATHER | \|\| id:ebi-a-GCST90001413 | MR Egger | 13.66039 | 17 | 0.690979 |
| 2 | ebi-a-GCST90001413 | finn-b-I9_CORATHER | Coronary atherosclerosis \|\| id:finn-b-I9_CORATHER | \|\| id:ebi-a-GCST90001413 | Inverse variance weighted | 15.34861 | 18 | 0.637907 |
| 3 | ebi-a-GCST90001422 | finn-b-I9_CORATHER | Coronary atherosclerosis \|\| id:finn-b-I9_CORATHER | \|\| id:ebi-a-GCST90001422 | MR Egger | 22.70423 | 22 | 0.418588 |
| 4 | ebi-a-GCST90001422 | finn-b-I9_CORATHER | Coronary atherosclerosis \|\| id:finn-b-I9_CORATHER | \|\| id:ebi-a-GCST90001422 | Inverse variance weighted | 25.64816 | 23 | 0.317755 |
| 5 | ebi-a-GCST90001446 | finn-b-I9_CORATHER | Coronary atherosclerosis \|\| id:finn-b-I9_CORATHER | \|\| id:ebi-a-GCST90001446 | MR Egger | 17.34847 | 14 | 0.238083 |
| 6 | ebi-a-GCST90001446 | finn-b-I9_CORATHER | Coronary atherosclerosis \|\| id:finn-b-I9_CORATHER | \|\| id:ebi-a-GCST90001446 | Inverse variance weighted | 17.37006 | 15 | 0.297227 |
| 7 | ebi-a-GCST90001447 | finn-b-I9_CORATHER | Coronary atherosclerosis \|\| id:finn-b-I9_CORATHER | \|\| id:ebi-a-GCST90001447 | MR Egger | 19.77894 | 24 | 0.709275 |
| 8 | ebi-a-GCST90001447 | finn-b-I9_CORATHER | Coronary atherosclerosis \|\| id:finn-b-I9_CORATHER | \|\| id:ebi-a-GCST90001447 | Inverse variance weighted | 19.77994 | 25 | 0.758272 |
| 9 | ebi-a-GCST90001464 | finn-b-I9_CORATHER | Coronary atherosclerosis \|\| id:finn-b-I9_CORATHER | \|\| id:ebi-a-GCST90001464 | MR Egger | 57.57587 | 65 | 0.731922 |
| 10 | ebi-a-GCST90001464 | finn-b-I9_CORATHER | Coronary atherosclerosis \|\| id:finn-b-I9_CORATHER | \|\| id:ebi-a-GCST90001464 | Inverse variance weighted | 57.74816 | 66 | 0.755488 |
| 11 | ebi-a-GCST90001471 | finn-b-I9_CORATHER | Coronary atherosclerosis \|\| id:finn-b-I9_CORATHER | \|\| id:ebi-a-GCST90001471 | MR Egger | 37.54066 | 34 | 0.310092 |
| 12 | ebi-a-GCST90001471 | finn-b-I9_CORATHER | Coronary atherosclerosis \|\| id:finn-b-I9_CORATHER | \|\| id:ebi-a-GCST90001471 | Inverse variance weighted | 38.73266 | 35 | 0.304919 |
| 13 | ebi-a-GCST90001473 | finn-b-I9_CORATHER | Coronary atherosclerosis \|\| id:finn-b-I9_CORATHER | \|\| id:ebi-a-GCST90001473 | MR Egger | 78.54599 | 84 | 0.647473 |
| 14 | ebi-a-GCST90001473 | finn-b-I9_CORATHER | Coronary atherosclerosis \|\| id:finn-b-I9_CORATHER | \|\| id:ebi-a-GCST90001473 | Inverse variance weighted | 79.46082 | 85 | 0.649136 |
| 15 | ebi-a-GCST90001514 | finn-b-I9_CORATHER | Coronary atherosclerosis \|\| id:finn-b-I9_CORATHER | \|\| id:ebi-a-GCST90001514 | MR Egger | 28.28109 | 25 | 0.295077 |
| 16 | ebi-a-GCST90001514 | finn-b-I9_CORATHER | Coronary atherosclerosis \|\| id:finn-b-I9_CORATHER | \|\| id:ebi-a-GCST90001514 | Inverse variance weighted | 30.68665 | 26 | 0.240146 |
| 17 | ebi-a-GCST90001559 | finn-b-I9_CORATHER | Coronary atherosclerosis \|\| id:finn-b-I9_CORATHER | \|\| id:ebi-a-GCST90001559 | MR Egger | 28.89781 | 30 | 0.52297 |
| 18 | ebi-a-GCST90001559 | finn-b-I9_CORATHER | Coronary atherosclerosis \|\| id:finn-b-I9_CORATHER | \|\| id:ebi-a-GCST90001559 | Inverse variance weighted | 29.02033 | 31 | 0.568175 |
| 19 | ebi-a-GCST90001576 | finn-b-I9_CORATHER | Coronary atherosclerosis \|\| id:finn-b-I9_CORATHER | \|\| id:ebi-a-GCST90001576 | MR Egger | 4.789062 | 15 | 0.993767 |
| 20 | ebi-a-GCST90001576 | finn-b-I9_CORATHER | Coronary atherosclerosis \|\| id:finn-b-I9_CORATHER | \|\| id:ebi-a-GCST90001576 | Inverse variance weighted | 8.953097 | 16 | 0.915332 |
| 21 | ebi-a-GCST90001587 | finn-b-I9_CORATHER | Coronary atherosclerosis \|\| id:finn-b-I9_CORATHER | \|\| id:ebi-a-GCST90001587 | MR Egger | 28.11421 | 38 | 0.879451 |
| 22 | ebi-a-GCST90001587 | finn-b-I9_CORATHER | Coronary atherosclerosis \|\| id:finn-b-I9_CORATHER | \|\| id:ebi-a-GCST90001587 | Inverse variance weighted | 28.56772 | 39 | 0.890544 |
| 23 | ebi-a-GCST90001588 | finn-b-I9_CORATHER | Coronary atherosclerosis \|\| id:finn-b-I9_CORATHER | \|\| id:ebi-a-GCST90001588 | MR Egger | 39.77533 | 33 | 0.193859 |
| 24 | ebi-a-GCST90001588 | finn-b-I9_CORATHER | Coronary atherosclerosis \|\| id:finn-b-I9_CORATHER | \|\| id:ebi-a-GCST90001588 | Inverse variance weighted | 39.78934 | 34 | 0.227946 |
| 25 | ebi-a-GCST90001598 | finn-b-I9_CORATHER | Coronary atherosclerosis \|\| id:finn-b-I9_CORATHER | \|\| id:ebi-a-GCST90001598 | MR Egger | 21.81473 | 24 | 0.590324 |
| 26 | ebi-a-GCST90001598 | finn-b-I9_CORATHER | Coronary atherosclerosis \|\| id:finn-b-I9_CORATHER | \|\| id:ebi-a-GCST90001598 | Inverse variance weighted | 23.32849 | 25 | 0.558388 |
| 27 | ebi-a-GCST90001613 | finn-b-I9_CORATHER | Coronary atherosclerosis \|\| id:finn-b-I9_CORATHER | \|\| id:ebi-a-GCST90001613 | MR Egger | 24.35952 | 27 | 0.610307 |
| 28 | ebi-a-GCST90001613 | finn-b-I9_CORATHER | Coronary atherosclerosis \|\| id:finn-b-I9_CORATHER | \|\| id:ebi-a-GCST90001613 | Inverse variance weighted | 28.10631 | 28 | 0.458825 |
| 29 | ebi-a-GCST90001648 | finn-b-I9_CORATHER | Coronary atherosclerosis \|\| id:finn-b-I9_CORATHER | \|\| id:ebi-a-GCST90001648 | MR Egger | 47.03574 | 59 | 0.869409 |
| 30 | ebi-a-GCST90001648 | finn-b-I9_CORATHER | Coronary atherosclerosis \|\| id:finn-b-I9_CORATHER | \|\| id:ebi-a-GCST90001648 | Inverse variance weighted | 47.65406 | 60 | 0.875578 |
| 31 | ebi-a-GCST90001649 | finn-b-I9_CORATHER | Coronary atherosclerosis \|\| id:finn-b-I9_CORATHER | \|\| id:ebi-a-GCST90001649 | MR Egger | 76.47364 | 63 | 0.118502 |
| 32 | ebi-a-GCST90001649 | finn-b-I9_CORATHER | Coronary atherosclerosis \|\| id:finn-b-I9_CORATHER | \|\| id:ebi-a-GCST90001649 | Inverse variance weighted | 76.47373 | 64 | 0.136506 |
| 33 | ebi-a-GCST90001650 | finn-b-I9_CORATHER | Coronary atherosclerosis \|\| id:finn-b-I9_CORATHER | \|\| id:ebi-a-GCST90001650 | MR Egger | 70.60342 | 64 | 0.266577 |
| 34 | ebi-a-GCST90001650 | finn-b-I9_CORATHER | Coronary atherosclerosis \|\| id:finn-b-I9_CORATHER | \|\| id:ebi-a-GCST90001650 | Inverse variance weighted | 70.75525 | 65 | 0.291609 |
| 35 | ebi-a-GCST90001682 | finn-b-I9_CORATHER | Coronary atherosclerosis \|\| id:finn-b-I9_CORATHER | \|\| id:ebi-a-GCST90001682 | MR Egger | 25.82731 | 24 | 0.361985 |
| 36 | ebi-a-GCST90001682 | finn-b-I9_CORATHER | Coronary atherosclerosis \|\| id:finn-b-I9_CORATHER | \|\| id:ebi-a-GCST90001682 | Inverse variance weighted | 29.10131 | 25 | 0.259703 |
| 37 | ebi-a-GCST90001904 | finn-b-I9_CORATHER | Coronary atherosclerosis \|\| id:finn-b-I9_CORATHER | \|\| id:ebi-a-GCST90001904 | MR Egger | 21.13185 | 21 | 0.450918 |
| 38 | ebi-a-GCST90001904 | finn-b-I9_CORATHER | Coronary atherosclerosis \|\| id:finn-b-I9_CORATHER | \|\| id:ebi-a-GCST90001904 | Inverse variance weighted | 21.98405 | 22 | 0.460841 |
| 39 | ebi-a-GCST90001923 | finn-b-I9_CORATHER | Coronary atherosclerosis \|\| id:finn-b-I9_CORATHER | \|\| id:ebi-a-GCST90001923 | MR Egger | 19.73826 | 17 | 0.287835 |
| 40 | ebi-a-GCST90001923 | finn-b-I9_CORATHER | Coronary atherosclerosis \|\| id:finn-b-I9_CORATHER | \|\| id:ebi-a-GCST90001923 | Inverse variance weighted | 20.00422 | 18 | 0.332582 |
| 41 | ebi-a-GCST90001926 | finn-b-I9_CORATHER | Coronary atherosclerosis \|\| id:finn-b-I9_CORATHER | \|\| id:ebi-a-GCST90001926 | MR Egger | 48.25149 | 48 | 0.462673 |
| 42 | ebi-a-GCST90001926 | finn-b-I9_CORATHER | Coronary atherosclerosis \|\| id:finn-b-I9_CORATHER | \|\| id:ebi-a-GCST90001926 | Inverse variance weighted | 49.35058 | 49 | 0.459101 |
| 43 | ebi-a-GCST90001931 | finn-b-I9_CORATHER | Coronary atherosclerosis \|\| id:finn-b-I9_CORATHER | \|\| id:ebi-a-GCST90001931 | MR Egger | 5.368339 | 7 | 0.615108 |
| 44 | ebi-a-GCST90001931 | finn-b-I9_CORATHER | Coronary atherosclerosis \|\| id:finn-b-I9_CORATHER | \|\| id:ebi-a-GCST90001931 | Inverse variance weighted | 5.724613 | 8 | 0.678048 |
| 45 | ebi-a-GCST90001935 | finn-b-I9_CORATHER | Coronary atherosclerosis \|\| id:finn-b-I9_CORATHER | \|\| id:ebi-a-GCST90001935 | MR Egger | 25.08029 | 21 | 0.243704 |
| 46 | ebi-a-GCST90001935 | finn-b-I9_CORATHER | Coronary atherosclerosis \|\| id:finn-b-I9_CORATHER | \|\| id:ebi-a-GCST90001935 | Inverse variance weighted | 25.81021 | 22 | 0.25992 |
| 47 | ebi-a-GCST90001966 | finn-b-I9_CORATHER | Coronary atherosclerosis \|\| id:finn-b-I9_CORATHER | \|\| id:ebi-a-GCST90001966 | MR Egger | 31.17153 | 30 | 0.40701 |
| 48 | ebi-a-GCST90001966 | finn-b-I9_CORATHER | Coronary atherosclerosis \|\| id:finn-b-I9_CORATHER | \|\| id:ebi-a-GCST90001966 | Inverse variance weighted | 31.87944 | 31 | 0.4226 |
| 49 | ebi-a-GCST90001969 | finn-b-I9_CORATHER | Coronary atherosclerosis \|\| id:finn-b-I9_CORATHER | \|\| id:ebi-a-GCST90001969 | MR Egger | 24.64078 | 23 | 0.369069 |
| 50 | ebi-a-GCST90001969 | finn-b-I9_CORATHER | Coronary atherosclerosis \|\| id:finn-b-I9_CORATHER | \|\| id:ebi-a-GCST90001969 | Inverse variance weighted | 27.76638 | 24 | 0.270017 |
| 51 | ebi-a-GCST90001980 | finn-b-I9_CORATHER | Coronary atherosclerosis \|\| id:finn-b-I9_CORATHER | \|\| id:ebi-a-GCST90001980 | MR Egger | 51.85155 | 53 | 0.518917 |
| 52 | ebi-a-GCST90001980 | finn-b-I9_CORATHER | Coronary atherosclerosis \|\| id:finn-b-I9_CORATHER | \|\| id:ebi-a-GCST90001980 | Inverse variance weighted | 53.66376 | 54 | 0.487309 |
| 53 | ebi-a-GCST90001981 | finn-b-I9_CORATHER | Coronary atherosclerosis \|\| id:finn-b-I9_CORATHER | \|\| id:ebi-a-GCST90001981 | MR Egger | 83.07524 | 75 | 0.244765 |
| 54 | ebi-a-GCST90001981 | finn-b-I9_CORATHER | Coronary atherosclerosis \|\| id:finn-b-I9_CORATHER | \|\| id:ebi-a-GCST90001981 | Inverse variance weighted | 83.50461 | 76 | 0.259974 |
| 55 | ebi-a-GCST90001989 | finn-b-I9_CORATHER | Coronary atherosclerosis \|\| id:finn-b-I9_CORATHER | \|\| id:ebi-a-GCST90001989 | MR Egger | 75.81399 | 92 | 0.88905 |
| 56 | ebi-a-GCST90001989 | finn-b-I9_CORATHER | Coronary atherosclerosis \|\| id:finn-b-I9_CORATHER | \|\| id:ebi-a-GCST90001989 | Inverse variance weighted | 76.14433 | 93 | 0.897948 |
| 57 | ebi-a-GCST90001993 | finn-b-I9_CORATHER | Coronary atherosclerosis \|\| id:finn-b-I9_CORATHER | \|\| id:ebi-a-GCST90001993 | MR Egger | 20.66995 | 16 | 0.191549 |
| 58 | ebi-a-GCST90001993 | finn-b-I9_CORATHER | Coronary atherosclerosis \|\| id:finn-b-I9_CORATHER | \|\| id:ebi-a-GCST90001993 | Inverse variance weighted | 20.7276 | 17 | 0.238713 |
| 59 | ebi-a-GCST90001994 | finn-b-I9_CORATHER | Coronary atherosclerosis \|\| id:finn-b-I9_CORATHER | \|\| id:ebi-a-GCST90001994 | MR Egger | 48.86628 | 41 | 0.186405 |
| 60 | ebi-a-GCST90001994 | finn-b-I9_CORATHER | Coronary atherosclerosis \|\| id:finn-b-I9_CORATHER | \|\| id:ebi-a-GCST90001994 | Inverse variance weighted | 48.86881 | 42 | 0.216527 |
| 61 | ebi-a-GCST90002012 | finn-b-I9_CORATHER | Coronary atherosclerosis \|\| id:finn-b-I9_CORATHER | \|\| id:ebi-a-GCST90002012 | MR Egger | 17.07339 | 25 | 0.879115 |
| 62 | ebi-a-GCST90002012 | finn-b-I9_CORATHER | Coronary atherosclerosis \|\| id:finn-b-I9_CORATHER | \|\| id:ebi-a-GCST90002012 | Inverse variance weighted | 17.49884 | 26 | 0.893239 |
| 63 | ebi-a-GCST90002017 | finn-b-I9_CORATHER | Coronary atherosclerosis \|\| id:finn-b-I9_CORATHER | \|\| id:ebi-a-GCST90002017 | MR Egger | 52.92661 | 40 | 0.082813 |
| 64 | ebi-a-GCST90002017 | finn-b-I9_CORATHER | Coronary atherosclerosis \|\| id:finn-b-I9_CORATHER | \|\| id:ebi-a-GCST90002017 | Inverse variance weighted | 53.26886 | 41 | 0.094872 |

**Table S3:** The horizontal pleiotropy test results for the causal relationships between 32 immune cell characteristics and coronary atherosclerosis.

|  | id.exposure | id.outcome | outcome | exposure | egger_intercept | se | pval |
| --- | --- | --- | --- | --- | --- | --- | --- |
| 1 | ebi-a-GCST90001413 | finn-b-I9_CORATHER | Coronary atherosclerosis \|\| id:finn-b-I9_CORATHER | \|\| id:ebi-a-GCST90001413 | 0.009358 | 0.007202 | 0.211181 |
| 2 | ebi-a-GCST90001422 | finn-b-I9_CORATHER | Coronary atherosclerosis \|\| id:finn-b-I9_CORATHER | \|\| id:ebi-a-GCST90001422 | -0.00955 | 0.005654 | 0.105351 |
| 3 | ebi-a-GCST90001446 | finn-b-I9_CORATHER | Coronary atherosclerosis \|\| id:finn-b-I9_CORATHER | \|\| id:ebi-a-GCST90001446 | 0.001636 | 0.012391 | 0.896854 |
| 4 | ebi-a-GCST90001447 | finn-b-I9_CORATHER | Coronary atherosclerosis \|\| id:finn-b-I9_CORATHER | \|\| id:ebi-a-GCST90001447 | -0.00026 | 0.008255 | 0.975082 |
| 5 | ebi-a-GCST90001464 | finn-b-I9_CORATHER | Coronary atherosclerosis \|\| id:finn-b-I9_CORATHER | \|\| id:ebi-a-GCST90001464 | 0.001567 | 0.003774 | 0.679453 |
| 6 | ebi-a-GCST90001471 | finn-b-I9_CORATHER | Coronary atherosclerosis \|\| id:finn-b-I9_CORATHER | \|\| id:ebi-a-GCST90001471 | -0.00549 | 0.005286 | 0.306125 |
| 7 | ebi-a-GCST90001473 | finn-b-I9_CORATHER | Coronary atherosclerosis \|\| id:finn-b-I9_CORATHER | \|\| id:ebi-a-GCST90001473 | -0.0037 | 0.003865 | 0.34158 |
| 8 | ebi-a-GCST90001514 | finn-b-I9_CORATHER | Coronary atherosclerosis \|\| id:finn-b-I9_CORATHER | \|\| id:ebi-a-GCST90001514 | -0.0089 | 0.006101 | 0.157223 |
| 9 | ebi-a-GCST90001559 | finn-b-I9_CORATHER | Coronary atherosclerosis \|\| id:finn-b-I9_CORATHER | \|\| id:ebi-a-GCST90001559 | -0.00203 | 0.005789 | 0.728769 |
| 10 | ebi-a-GCST90001576 | finn-b-I9_CORATHER | Coronary atherosclerosis \|\| id:finn-b-I9_CORATHER | \|\| id:ebi-a-GCST90001576 | -0.01939 | 0.009501 | 0.059299 |
| 11 | ebi-a-GCST90001587 | finn-b-I9_CORATHER | Coronary atherosclerosis \|\| id:finn-b-I9_CORATHER | \|\| id:ebi-a-GCST90001587 | -0.00452 | 0.006712 | 0.504747 |
| 12 | ebi-a-GCST90001588 | finn-b-I9_CORATHER | Coronary atherosclerosis \|\| id:finn-b-I9_CORATHER | \|\| id:ebi-a-GCST90001588 | -0.00063 | 0.005822 | 0.914802 |
| 13 | ebi-a-GCST90001598 | finn-b-I9_CORATHER | Coronary atherosclerosis \|\| id:finn-b-I9_CORATHER | \|\| id:ebi-a-GCST90001598 | -0.0094 | 0.007639 | 0.230489 |
| 14 | ebi-a-GCST90001613 | finn-b-I9_CORATHER | Coronary atherosclerosis \|\| id:finn-b-I9_CORATHER | \|\| id:ebi-a-GCST90001613 | -0.00828 | 0.004275 | 0.063445 |
| 15 | ebi-a-GCST90001648 | finn-b-I9_CORATHER | Coronary atherosclerosis \|\| id:finn-b-I9_CORATHER | \|\| id:ebi-a-GCST90001648 | -0.00437 | 0.005558 | 0.434817 |
| 16 | ebi-a-GCST90001649 | finn-b-I9_CORATHER | Coronary atherosclerosis \|\| id:finn-b-I9_CORATHER | \|\| id:ebi-a-GCST90001649 | ###### | 0.005162 | 0.993222 |
| 17 | ebi-a-GCST90001650 | finn-b-I9_CORATHER | Coronary atherosclerosis \|\| id:finn-b-I9_CORATHER | \|\| id:ebi-a-GCST90001650 | 0.001825 | 0.004919 | 0.711876 |
| 18 | ebi-a-GCST90001682 | finn-b-I9_CORATHER | Coronary atherosclerosis \|\| id:finn-b-I9_CORATHER | \|\| id:ebi-a-GCST90001682 | 0.017323 | 0.009932 | 0.093914 |
| 19 | ebi-a-GCST90001904 | finn-b-I9_CORATHER | Coronary atherosclerosis \|\| id:finn-b-I9_CORATHER | \|\| id:ebi-a-GCST90001904 | -0.00586 | 0.006372 | 0.367888 |
| 20 | ebi-a-GCST90001923 | finn-b-I9_CORATHER | Coronary atherosclerosis \|\| id:finn-b-I9_CORATHER | \|\| id:ebi-a-GCST90001923 | 0.002863 | 0.005982 | 0.638314 |
| 21 | ebi-a-GCST90001926 | finn-b-I9_CORATHER | Coronary atherosclerosis \|\| id:finn-b-I9_CORATHER | \|\| id:ebi-a-GCST90001926 | 0.005014 | 0.004795 | 0.300966 |
| 22 | ebi-a-GCST90001931 | finn-b-I9_CORATHER | Coronary atherosclerosis \|\| id:finn-b-I9_CORATHER | \|\| id:ebi-a-GCST90001931 | 0.008819 | 0.014775 | 0.569376 |
| 23 | ebi-a-GCST90001935 | finn-b-I9_CORATHER | Coronary atherosclerosis \|\| id:finn-b-I9_CORATHER | \|\| id:ebi-a-GCST90001935 | 0.006737 | 0.008618 | 0.443071 |
| 24 | ebi-a-GCST90001966 | finn-b-I9_CORATHER | Coronary atherosclerosis \|\| id:finn-b-I9_CORATHER | \|\| id:ebi-a-GCST90001966 | -0.00515 | 0.006242 | 0.415654 |
| 25 | ebi-a-GCST90001969 | finn-b-I9_CORATHER | Coronary atherosclerosis \|\| id:finn-b-I9_CORATHER | \|\| id:ebi-a-GCST90001969 | -0.01155 | 0.006763 | 0.101089 |
| 26 | ebi-a-GCST90001980 | finn-b-I9_CORATHER | Coronary atherosclerosis \|\| id:finn-b-I9_CORATHER | \|\| id:ebi-a-GCST90001980 | -0.00694 | 0.005154 | 0.183973 |
| 27 | ebi-a-GCST90001981 | finn-b-I9_CORATHER | Coronary atherosclerosis \|\| id:finn-b-I9_CORATHER | \|\| id:ebi-a-GCST90001981 | -0.00249 | 0.004002 | 0.535433 |
| 28 | ebi-a-GCST90001989 | finn-b-I9_CORATHER | Coronary atherosclerosis \|\| id:finn-b-I9_CORATHER | \|\| id:ebi-a-GCST90001989 | -0.00195 | 0.003393 | 0.566865 |
| 29 | ebi-a-GCST90001993 | finn-b-I9_CORATHER | Coronary atherosclerosis \|\| id:finn-b-I9_CORATHER | \|\| id:ebi-a-GCST90001993 | 0.002288 | 0.010829 | 0.835371 |
| 30 | ebi-a-GCST90001994 | finn-b-I9_CORATHER | Coronary atherosclerosis \|\| id:finn-b-I9_CORATHER | \|\| id:ebi-a-GCST90001994 | 0.000203 | 0.004408 | 0.963458 |
| 31 | ebi-a-GCST90002012 | finn-b-I9_CORATHER | Coronary atherosclerosis \|\| id:finn-b-I9_CORATHER | \|\| id:ebi-a-GCST90002012 | 0.003446 | 0.005284 | 0.520183 |
| 32 | ebi-a-GCST90002017 | finn-b-I9_CORATHER | Coronary atherosclerosis \|\| id:finn-b-I9_CORATHER | \|\| id:ebi-a-GCST90002017 | 0.003842 | 0.007555 | 0.613837 |

**Table S4:** Reverse Mendelian randomization analysis of the causal relationship between 32 immune cell traits and coronary atherosclerosis.

|  | id.exposure | id.outcome | outcome | exposure | method | nsnp | b | se | pval | lo_ci | up_ci | or | or_lci95 | or_uci95 |
| --- | --- | --- | --- | --- | --- | --- | --- | --- | --- | --- | --- | --- | --- | --- |
| 1 | finn-b-I9_CORATHER | ebi-a-GCST90001413 | IgD- CD24- B cell %B cell \|\| id:ebi-a-GCST90001413 | \|\| id:finn-b-I9_CORATHER | Inverse variance weighted | 184 | -0.02017 | 0.025232 | 0.424007 | -0.06963 | 0.029282 | 0.980029 | 0.932741 | 1.029715 |
| 2 | finn-b-I9_CORATHER | ebi-a-GCST90001422 | CD20- CD38- B cell %B cell \|\| id:ebi-a-GCST90001422 | \|\| id:finn-b-I9_CORATHER | Inverse variance weighted | 184 | 0.043488 | 0.024844 | 0.080044 | -0.00521 | 0.092182 | 1.044447 | 0.994807 | 1.096564 |
| 3 | finn-b-I9_CORATHER | ebi-a-GCST90001446 | IgD- CD38- B cell Absolute Count \|\| id:ebi-a-GCST90001446 | \|\| id:finn-b-I9_CORATHER | Inverse variance weighted | 184 | -0.01864 | 0.025505 | 0.464764 | -0.06863 | 0.031345 | 0.981528 | 0.933667 | 1.031842 |
| 4 | finn-b-I9_CORATHER | ebi-a-GCST90001447 | IgD+ CD38+ B cell %B cell \|\| id:ebi-a-GCST90001447 | \|\| id:finn-b-I9_CORATHER | Inverse variance weighted | 184 | 0.027851 | 0.025092 | 0.267026 | -0.02133 | 0.077032 | 1.028242 | 0.978896 | 1.080077 |
| 5 | finn-b-I9_CORATHER | ebi-a-GCST90001464 | CD86+ myeloid Dendritic Cell Absolute Count \|\| id:ebi-a-GCST90001464 | \|\| id:finn-b-I9_CORATHER | Inverse variance weighted | 184 | -0.03809 | 0.026633 | 0.152628 | -0.09029 | 0.014107 | 0.962624 | 0.913664 | 1.014207 |
| 6 | finn-b-I9_CORATHER | ebi-a-GCST90001471 | CD62L- plasmacytoid Dendritic Cell %Dendritic Cell \|\| id:ebi-a-GCST90001471 | \|\| id:finn-b-I9_CORATHER | Inverse variance weighted | 184 | 0.05129 | 0.026046 | 0.048928 | 0.00024 | 0.102341 | 1.052629 | 1.00024 | 1.107761 |
| 7 | finn-b-I9_CORATHER | ebi-a-GCST90001473 | CD62L- CD86+ myeloid Dendritic Cell %Dendritic Cell \|\| id:ebi-a-GCST90001473 | \|\| id:finn-b-I9_CORATHER | Inverse variance weighted | 180 | -0.01725 | 0.026786 | 0.519463 | -0.06975 | 0.035245 | 0.982893 | 0.932623 | 1.035874 |
| 8 | finn-b-I9_CORATHER | ebi-a-GCST90001514 | Hematopoietic Stem Cell Absolute Count \|\| id:ebi-a-GCST90001514 | \|\| id:finn-b-I9_CORATHER | Inverse variance weighted | 183 | 0.001727 | 0.032512 | 0.957646 | -0.062 | 0.065449 | 1.001728 | 0.939886 | 1.067639 |
| 9 | finn-b-I9_CORATHER | ebi-a-GCST90001559 | Terminally Differentiated CD8+ T cell %T cell \|\| id:ebi-a-GCST90001559 | \|\| id:finn-b-I9_CORATHER | Inverse variance weighted | 184 | 0.016838 | 0.025651 | 0.511564 | -0.03344 | 0.067114 | 1.01698 | 0.967114 | 1.069417 |
| 10 | finn-b-I9_CORATHER | ebi-a-GCST90001576 | Transitional B cell %B cell \|\| id:ebi-a-GCST90001576 | \|\| id:finn-b-I9_CORATHER | Inverse variance weighted | 184 | 0.026856 | 0.025742 | 0.296828 | -0.0236 | 0.07731 | 1.02722 | 0.976677 | 1.080377 |
| 11 | finn-b-I9_CORATHER | ebi-a-GCST90001587 | CD16+ monocyte %monocyte \|\| id:ebi-a-GCST90001587 | \|\| id:finn-b-I9_CORATHER | Inverse variance weighted | 184 | -0.01741 | 0.0247 | 0.480809 | -0.06583 | 0.030999 | 0.982737 | 0.936294 | 1.031484 |
| 12 | finn-b-I9_CORATHER | ebi-a-GCST90001588 | T/B cell \|\| id:ebi-a-GCST90001588 | \|\| id:finn-b-I9_CORATHER | Inverse variance weighted | 184 | 0.03006 | 0.025857 | 0.245015 | -0.02062 | 0.08074 | 1.030516 | 0.979591 | 1.084089 |
| 13 | finn-b-I9_CORATHER | ebi-a-GCST90001598 | CD4-CD8- T cell Absolute Count \|\| id:ebi-a-GCST90001598 | \|\| id:finn-b-I9_CORATHER | Inverse variance weighted | 184 | 0.012058 | 0.023808 | 0.612545 | -0.03461 | 0.058722 | 1.012131 | 0.965985 | 1.06048 |
| 14 | finn-b-I9_CORATHER | ebi-a-GCST90001613 | CD4-CD8- T cell %leukocyte \|\| id:ebi-a-GCST90001613 | \|\| id:finn-b-I9_CORATHER | Inverse variance weighted | 184 | 0.01114 | 0.024206 | 0.645355 | -0.0363 | 0.058585 | 1.011203 | 0.964347 | 1.060335 |
| 15 | finn-b-I9_CORATHER | ebi-a-GCST90001648 | HLA DR+ Natural Killer Absolute Count \|\| id:ebi-a-GCST90001648 | \|\| id:finn-b-I9_CORATHER | Inverse variance weighted | 184 | 0.028501 | 0.025145 | 0.257022 | -0.02078 | 0.077785 | 1.028911 | 0.979431 | 1.080891 |
| 16 | finn-b-I9_CORATHER | ebi-a-GCST90001649 | HLA DR+ Natural Killer %Natural Killer \|\| id:ebi-a-GCST90001649 | \|\| id:finn-b-I9_CORATHER | Inverse variance weighted | 184 | 0.036078 | 0.025663 | 0.15978 | -0.01422 | 0.086377 | 1.036736 | 0.985878 | 1.090218 |
| 17 | finn-b-I9_CORATHER | ebi-a-GCST90001650 | HLA DR+ Natural Killer %CD3- lymphocyte \|\| id:ebi-a-GCST90001650 | \|\| id:finn-b-I9_CORATHER | Inverse variance weighted | 184 | 0.03468 | 0.025162 | 0.168116 | -0.01464 | 0.083998 | 1.035289 | 0.985469 | 1.087627 |
| 18 | finn-b-I9_CORATHER | ebi-a-GCST90001682 | CD127- CD8+ T cell %T cell \|\| id:ebi-a-GCST90001682 | \|\| id:finn-b-I9_CORATHER | Inverse variance weighted | 184 | 0.016295 | 0.025461 | 0.522175 | -0.03361 | 0.066197 | 1.016428 | 0.96695 | 1.068438 |
| 19 | finn-b-I9_CORATHER | ebi-a-GCST90001904 | CD86 on CD62L+ myeloid Dendritic Cell \|\| id:ebi-a-GCST90001904 | \|\| id:finn-b-I9_CORATHER | Inverse variance weighted | 184 | 0.01147 | 0.027893 | 0.680914 | -0.0432 | 0.06614 | 1.011536 | 0.95772 | 1.068376 |
| 20 | finn-b-I9_CORATHER | ebi-a-GCST90001923 | CD127 on CD45RA- CD4 not regulatory T cell \|\| id:ebi-a-GCST90001923 | \|\| id:finn-b-I9_CORATHER | Inverse variance weighted | 184 | 0.061097 | 0.028962 | 0.034897 | 0.004331 | 0.117862 | 1.063002 | 1.004341 | 1.125089 |
| 21 | finn-b-I9_CORATHER | ebi-a-GCST90001926 | CD127 on granulocyte \|\| id:ebi-a-GCST90001926 | \|\| id:finn-b-I9_CORATHER | Inverse variance weighted | 184 | -0.00976 | 0.029253 | 0.738754 | -0.06709 | 0.04758 | 0.990291 | 0.935109 | 1.04873 |
| 22 | finn-b-I9_CORATHER | ebi-a-GCST90001931 | CD127 on CD4+ T cell \|\| id:ebi-a-GCST90001931 | \|\| id:finn-b-I9_CORATHER | Inverse variance weighted | 55 | 0.028274 | 0.052999 | 0.593695 | -0.0756 | 0.132151 | 1.028678 | 0.927184 | 1.141281 |
| 23 | finn-b-I9_CORATHER | ebi-a-GCST90001935 | CD25 on CD39+ CD4 regulatory T cell \|\| id:ebi-a-GCST90001935 | \|\| id:finn-b-I9_CORATHER | Inverse variance weighted | 184 | 0.007771 | 0.026488 | 0.769228 | -0.04415 | 0.059688 | 1.007801 | 0.956815 | 1.061505 |
| 24 | finn-b-I9_CORATHER | ebi-a-GCST90001966 | FSC-A on granulocyte \|\| id:ebi-a-GCST90001966 | \|\| id:finn-b-I9_CORATHER | Inverse variance weighted | 184 | 0.001941 | 0.028519 | 0.945751 | -0.05396 | 0.057838 | 1.001942 | 0.947473 | 1.059543 |
| 25 | finn-b-I9_CORATHER | ebi-a-GCST90001969 | FSC-A on Natural Killer \|\| id:ebi-a-GCST90001969 | \|\| id:finn-b-I9_CORATHER | Inverse variance weighted | 184 | -0.02198 | 0.028052 | 0.433284 | -0.07696 | 0.033001 | 0.978259 | 0.925924 | 1.033551 |
| 26 | finn-b-I9_CORATHER | ebi-a-GCST90001980 | CD40 on CD14+ CD16- monocyte \|\| id:ebi-a-GCST90001980 | \|\| id:finn-b-I9_CORATHER | Inverse variance weighted | 184 | 0.008211 | 0.027546 | 0.765636 | -0.04578 | 0.062202 | 1.008245 | 0.955252 | 1.064178 |
| 27 | finn-b-I9_CORATHER | ebi-a-GCST90001981 | CD40 on CD14+ CD16+ monocyte \|\| id:ebi-a-GCST90001981 | \|\| id:finn-b-I9_CORATHER | Inverse variance weighted | 184 | 0.053096 | 0.027711 | 0.055351 | -0.00122 | 0.107409 | 1.054531 | 0.998785 | 1.11339 |
| 28 | finn-b-I9_CORATHER | ebi-a-GCST90001989 | CD40 on CD14- CD16+ monocyte \|\| id:ebi-a-GCST90001989 | \|\| id:finn-b-I9_CORATHER | Inverse variance weighted | 184 | 0.048388 | 0.029094 | 0.096281 | -0.00864 | 0.105413 | 1.049578 | 0.991401 | 1.11117 |
| 29 | finn-b-I9_CORATHER | ebi-a-GCST90001993 | PDL-1 on CD14+ CD16- monocyte \|\| id:ebi-a-GCST90001993 | \|\| id:finn-b-I9_CORATHER | Inverse variance weighted | 184 | 0.008331 | 0.025096 | 0.739912 | -0.04086 | 0.05752 | 1.008366 | 0.959966 | 1.059206 |
| 30 | finn-b-I9_CORATHER | ebi-a-GCST90001994 | CX3CR1 on CD14- CD16- \|\| id:ebi-a-GCST90001994 | \|\| id:finn-b-I9_CORATHER | Inverse variance weighted | 184 | 0.010966 | 0.025805 | 0.67087 | -0.03961 | 0.061543 | 1.011026 | 0.961163 | 1.063476 |
| 31 | finn-b-I9_CORATHER | ebi-a-GCST90002012 | CX3CR1 on CD14- CD16+ monocyte \|\| id:ebi-a-GCST90002012 | \|\| id:finn-b-I9_CORATHER | Inverse variance weighted | 184 | -0.04017 | 0.024732 | 0.104339 | -0.08864 | 0.008306 | 0.960627 | 0.915172 | 1.00834 |
| 32 | finn-b-I9_CORATHER | ebi-a-GCST90002017 | CCR2 on monocyte \|\| id:ebi-a-GCST90002017 | \|\| id:finn-b-I9_CORATHER | Inverse variance weighted | 184 | -0.0093 | 0.028218 | 0.741792 | -0.0646 | 0.046009 | 0.990746 | 0.937439 | 1.047084 |

**Table S5:** Mendelian randomization analysis of the causal relationship between fatty acid levels and coronary atherosclerosis.

|  | id.exposure | id.outcome | outcome | exposure | method | nsnp | b | se | pval | lo_ci | up_ci | or | or_lci95 | or_uci95 |
| --- | --- | --- | --- | --- | --- | --- | --- | --- | --- | --- | --- | --- | --- | --- |
| 1 | ebi-a-GCST90092987 | finn-b-I9_CORATHER | Coronary atherosclerosis \|\| id:finn-b-I9_CORATHER | \|\| id:ebi-a-GCST90092987 | Inverse variance weighted | 424 | 0.227527 | 0.036976 | 7.59E-10 | 0.155054 | 0.3 | 1.255492 | 1.167721 | 1.349859 |

**Table S6:** Reverse Mendelian randomization analysis of the causal relationship between fatty acid levels and coronary atherosclerosis.

|  | id.exposure | id.outcome | outcome | exposure | method | nsnp | b | se | pval | lo_ci | up_ci | or | or_lci95 | or_uci95 |
| --- | --- | --- | --- | --- | --- | --- | --- | --- | --- | --- | --- | --- | --- | --- |
| 1 | finn-b-I9_CORATHER | ebi-a-GCST90092987 | Total fatty acid levels \|\| id:ebi-a-GCST90092987 | \|\| id:finn-b-I9_CORATHER | Inverse variance weighted | 184 | -0.00493 | 0.015672 | 0.753112 | -0.03565 | 0.025788 | 0.995083 | 0.96498 | 1.026124 |

**Table S7:** Horizontal pleiotropy test for fatty acid levels and coronary atherosclerosis.

|  | id.exposure | id.outcome | outcome | exposure | egger_intercept | se | pval |
| --- | --- | --- | --- | --- | --- | --- | --- |
| 1 | ebi-a-GCST90092987 | finn-b-I9_CORATHER | Coronary atherosclerosis \|\| id:finn-b-I9_CORATHER | \|\| id:ebi-a-GCST90092987 | -0.00057 | 0.002807 | 0.83982 |

**Table S8:** Mendelian randomization analysis of the causal relationship between fatty acid levels and immune cell traits.

|  | id.exposure | id.outcome | outcome | exposure | method | nsnp | b | se | pval | lo_ci | up_ci | or | or_lci95 | or_uci95 |
| --- | --- | --- | --- | --- | --- | --- | --- | --- | --- | --- | --- | --- | --- | --- |
| 1 | ebi-a-GCST90092980 | ebi-a-GCST90001413 | IgD- CD24- B cell %B cell \|\| id:ebi-a-GCST90001413 | \|\| id:ebi-a-GCST90092980 | Inverse variance weighted | 173 | -0.02851 | 0.074044 | 0.700218 | -0.17363 | 0.116617 | 0.971894 | 0.840604 | 1.123689 |
| 2 | ebi-a-GCST90092980 | ebi-a-GCST90001422 | CD20- CD38- B cell %B cell \|\| id:ebi-a-GCST90001422 | \|\| id:ebi-a-GCST90092980 | Inverse variance weighted | 173 | -0.04357 | 0.065335 | 0.504833 | -0.17163 | 0.084484 | 0.957363 | 0.842292 | 1.088156 |
| 3 | ebi-a-GCST90092980 | ebi-a-GCST90001446 | IgD- CD38- B cell Absolute Count \|\| id:ebi-a-GCST90001446 | \|\| id:ebi-a-GCST90092980 | Inverse variance weighted | 173 | -0.09104 | 0.067406 | 0.176795 | -0.22316 | 0.041071 | 0.912977 | 0.799986 | 1.041926 |
| 4 | ebi-a-GCST90092980 | ebi-a-GCST90001447 | IgD+ CD38+ B cell %B cell \|\| id:ebi-a-GCST90001447 | \|\| id:ebi-a-GCST90092980 | Inverse variance weighted | 173 | -0.02433 | 0.068006 | 0.720516 | -0.15762 | 0.108961 | 0.975963 | 0.854173 | 1.115119 |
| 5 | ebi-a-GCST90092980 | ebi-a-GCST90001464 | CD86+ myeloid Dendritic Cell Absolute Count \|\| id:ebi-a-GCST90001464 | \|\| id:ebi-a-GCST90092980 | Inverse variance weighted | 173 | 0.104937 | 0.070283 | 0.13542 | -0.03282 | 0.24269 | 1.11064 | 0.967715 | 1.274674 |
| 6 | ebi-a-GCST90092980 | ebi-a-GCST90001473 | CD62L- CD86+ myeloid Dendritic Cell %Dendritic Cell \|\| id:ebi-a-GCST90001473 | \|\| id:ebi-a-GCST90092980 | Inverse variance weighted | 168 | 0.140713 | 0.071382 | 0.048695 | 0.000803 | 0.280622 | 1.151094 | 1.000803 | 1.323953 |
| 7 | ebi-a-GCST90092980 | ebi-a-GCST90001514 | Hematopoietic Stem Cell Absolute Count \|\| id:ebi-a-GCST90001514 | \|\| id:ebi-a-GCST90092980 | Inverse variance weighted | 173 | 0.073839 | 0.08796 | 0.401209 | -0.09856 | 0.24624 | 1.076633 | 0.90614 | 1.279206 |
| 8 | ebi-a-GCST90092980 | ebi-a-GCST90001559 | Terminally Differentiated CD8+ T cell %T cell \|\| id:ebi-a-GCST90001559 | \|\| id:ebi-a-GCST90092980 | Inverse variance weighted | 173 | 0.002529 | 0.067347 | 0.97004 | -0.12947 | 0.134529 | 1.002533 | 0.878561 | 1.143998 |
| 9 | ebi-a-GCST90092980 | ebi-a-GCST90001576 | Transitional B cell %B cell \|\| id:ebi-a-GCST90001576 | \|\| id:ebi-a-GCST90092980 | Inverse variance weighted | 173 | -0.1152 | 0.073183 | 0.115459 | -0.25864 | 0.028239 | 0.891189 | 0.772104 | 1.028642 |
| 10 | ebi-a-GCST90092980 | ebi-a-GCST90001587 | CD16+ monocyte %monocyte \|\| id:ebi-a-GCST90001587 | \|\| id:ebi-a-GCST90092980 | Inverse variance weighted | 173 | 0.001876 | 0.065193 | 0.977039 | -0.1259 | 0.129656 | 1.001878 | 0.8817 | 1.138436 |
| 11 | ebi-a-GCST90092980 | ebi-a-GCST90001588 | T/B cell \|\| id:ebi-a-GCST90001588 | \|\| id:ebi-a-GCST90092980 | Inverse variance weighted | 173 | 0.087642 | 0.066478 | 0.187383 | -0.04265 | 0.217938 | 1.091597 | 0.958242 | 1.24351 |
| 12 | ebi-a-GCST90092980 | ebi-a-GCST90001598 | CD4-CD8- T cell Absolute Count \|\| id:ebi-a-GCST90001598 | \|\| id:ebi-a-GCST90092980 | Inverse variance weighted | 173 | 0.019043 | 0.062741 | 0.761498 | -0.10393 | 0.142015 | 1.019225 | 0.901289 | 1.152593 |
| 13 | ebi-a-GCST90092980 | ebi-a-GCST90001613 | CD4-CD8- T cell %leukocyte \|\| id:ebi-a-GCST90001613 | \|\| id:ebi-a-GCST90092980 | Inverse variance weighted | 173 | -0.009 | 0.063814 | 0.887904 | -0.13407 | 0.116081 | 0.991045 | 0.874528 | 1.123086 |
| 14 | ebi-a-GCST90092980 | ebi-a-GCST90001648 | HLA DR+ Natural Killer Absolute Count \|\| id:ebi-a-GCST90001648 | \|\| id:ebi-a-GCST90092980 | Inverse variance weighted | 173 | 0.036469 | 0.066358 | 0.582607 | -0.09359 | 0.166531 | 1.037142 | 0.910654 | 1.1812 |
| 15 | ebi-a-GCST90092980 | ebi-a-GCST90001649 | HLA DR+ Natural Killer %Natural Killer \|\| id:ebi-a-GCST90001649 | \|\| id:ebi-a-GCST90092980 | Inverse variance weighted | 173 | 0.095958 | 0.067843 | 0.15724 | -0.03701 | 0.228931 | 1.100713 | 0.963663 | 1.257255 |
| 16 | ebi-a-GCST90092980 | ebi-a-GCST90001650 | HLA DR+ Natural Killer %CD3- lymphocyte \|\| id:ebi-a-GCST90001650 | \|\| id:ebi-a-GCST90092980 | Inverse variance weighted | 173 | 0.053654 | 0.066518 | 0.419887 | -0.07672 | 0.184029 | 1.05512 | 0.926149 | 1.20205 |
| 17 | ebi-a-GCST90092980 | ebi-a-GCST90001682 | CD127- CD8+ T cell %T cell \|\| id:ebi-a-GCST90001682 | \|\| id:ebi-a-GCST90092980 | Inverse variance weighted | 173 | 0.048737 | 0.069426 | 0.482681 | -0.08734 | 0.184811 | 1.049944 | 0.916368 | 1.202991 |
| 18 | ebi-a-GCST90092980 | ebi-a-GCST90001685 | CD28- CD8+ T cell %T cell \|\| id:ebi-a-GCST90001685 | \|\| id:ebi-a-GCST90092980 | Inverse variance weighted | 173 | 0.062472 | 0.067153 | 0.35222 | -0.06915 | 0.194093 | 1.064465 | 0.933188 | 1.214209 |
| 19 | ebi-a-GCST90092980 | ebi-a-GCST90001686 | CD28- CD8+ T cell %CD8+ T cell \|\| id:ebi-a-GCST90001686 | \|\| id:ebi-a-GCST90092980 | Inverse variance weighted | 173 | 0.060302 | 0.06383 | 0.344802 | -0.06481 | 0.185409 | 1.062157 | 0.93725 | 1.20371 |
| 20 | ebi-a-GCST90092980 | ebi-a-GCST90001687 | CD28- CD8+ T cell Absolute Count \|\| id:ebi-a-GCST90001687 | \|\| id:ebi-a-GCST90092980 | Inverse variance weighted | 173 | 0.08802 | 0.071131 | 0.215921 | -0.0514 | 0.227437 | 1.09201 | 0.949903 | 1.255378 |
| 21 | ebi-a-GCST90092980 | ebi-a-GCST90001888 | CD28 on CD39+ secreting CD4 regulatory T cell \|\| id:ebi-a-GCST90001888 | \|\| id:ebi-a-GCST90092980 | Inverse variance weighted | 173 | -0.02744 | 0.072195 | 0.703869 | -0.16894 | 0.114061 | 0.972932 | 0.844556 | 1.12082 |
| 22 | ebi-a-GCST90092980 | ebi-a-GCST90001900 | CD28 on resting CD4 regulatory T cell \|\| id:ebi-a-GCST90001900 | \|\| id:ebi-a-GCST90092980 | Inverse variance weighted | 41 | 0.086065 | 0.143917 | 0.549827 | -0.19601 | 0.368142 | 1.089877 | 0.822002 | 1.445048 |
| 23 | ebi-a-GCST90092980 | ebi-a-GCST90001902 | CD28 on activated CD4 regulatory T cell \|\| id:ebi-a-GCST90001902 | \|\| id:ebi-a-GCST90092980 | Inverse variance weighted | 173 | -0.00543 | 0.06878 | 0.93709 | -0.14024 | 0.129381 | 0.994586 | 0.869151 | 1.138123 |
| 24 | ebi-a-GCST90092980 | ebi-a-GCST90001904 | CD86 on CD62L+ myeloid Dendritic Cell \|\| id:ebi-a-GCST90001904 | \|\| id:ebi-a-GCST90092980 | Inverse variance weighted | 173 | 0.008932 | 0.072762 | 0.902305 | -0.13368 | 0.151546 | 1.008972 | 0.874868 | 1.163632 |
| 25 | ebi-a-GCST90092980 | ebi-a-GCST90001914 | CD45 on lymphocyte \|\| id:ebi-a-GCST90001914 | \|\| id:ebi-a-GCST90092980 | Inverse variance weighted | 173 | 0.011627 | 0.069524 | 0.867179 | -0.12464 | 0.147894 | 1.011695 | 0.882815 | 1.159391 |
| 26 | ebi-a-GCST90092980 | ebi-a-GCST90001926 | CD127 on granulocyte \|\| id:ebi-a-GCST90001926 | \|\| id:ebi-a-GCST90092980 | Inverse variance weighted | 173 | 0.038799 | 0.079938 | 0.627416 | -0.11788 | 0.195477 | 1.039562 | 0.888804 | 1.215891 |
| 27 | ebi-a-GCST90092980 | ebi-a-GCST90001931 | CD127 on CD4+ T cell \|\| id:ebi-a-GCST90001931 | \|\| id:ebi-a-GCST90092980 | Inverse variance weighted | 39 | -0.18273 | 0.161969 | 0.259254 | -0.50018 | 0.134733 | 0.832996 | 0.606419 | 1.144231 |
| 28 | ebi-a-GCST90092980 | ebi-a-GCST90001934 | CD25 on CD45RA+ CD4 not regulatory T cell \|\| id:ebi-a-GCST90001934 | \|\| id:ebi-a-GCST90092980 | Inverse variance weighted | 173 | 0.087369 | 0.064182 | 0.17343 | -0.03843 | 0.213167 | 1.0913 | 0.962301 | 1.237591 |
| 29 | ebi-a-GCST90092980 | ebi-a-GCST90001935 | CD25 on CD39+ CD4 regulatory T cell \|\| id:ebi-a-GCST90001935 | \|\| id:ebi-a-GCST90092980 | Inverse variance weighted | 173 | 0.014124 | 0.067173 | 0.833461 | -0.11754 | 0.145783 | 1.014224 | 0.889109 | 1.156946 |
| 30 | ebi-a-GCST90092980 | ebi-a-GCST90001966 | FSC-A on granulocyte \|\| id:ebi-a-GCST90001966 | \|\| id:ebi-a-GCST90092980 | Inverse variance weighted | 173 | 0.019162 | 0.080158 | 0.811066 | -0.13795 | 0.176271 | 1.019347 | 0.871145 | 1.192761 |
| 31 | ebi-a-GCST90092980 | ebi-a-GCST90001969 | FSC-A on Natural Killer \|\| id:ebi-a-GCST90001969 | \|\| id:ebi-a-GCST90092980 | Inverse variance weighted | 173 | 0.081649 | 0.07037 | 0.245932 | -0.05628 | 0.219575 | 1.085075 | 0.945278 | 1.245547 |
| 32 | ebi-a-GCST90092980 | ebi-a-GCST90001980 | CD40 on CD14+ CD16- monocyte \|\| id:ebi-a-GCST90001980 | \|\| id:ebi-a-GCST90092980 | Inverse variance weighted | 173 | -0.04994 | 0.068363 | 0.465068 | -0.18393 | 0.084051 | 0.951285 | 0.831991 | 1.087684 |
| 33 | ebi-a-GCST90092980 | ebi-a-GCST90001981 | CD40 on CD14+ CD16+ monocyte \|\| id:ebi-a-GCST90001981 | \|\| id:ebi-a-GCST90092980 | Inverse variance weighted | 173 | -0.02326 | 0.071231 | 0.743984 | -0.16288 | 0.11635 | 0.977006 | 0.849697 | 1.123389 |
| 34 | ebi-a-GCST90092980 | ebi-a-GCST90001984 | HLA DR on CD14- CD16+ monocyte \|\| id:ebi-a-GCST90001984 | \|\| id:ebi-a-GCST90092980 | Inverse variance weighted | 173 | 0.11339 | 0.074917 | 0.130142 | -0.03345 | 0.260227 | 1.120069 | 0.967106 | 1.297225 |
| 35 | ebi-a-GCST90092980 | ebi-a-GCST90001989 | CD40 on CD14- CD16+ monocyte \|\| id:ebi-a-GCST90001989 | \|\| id:ebi-a-GCST90092980 | Inverse variance weighted | 173 | -0.01064 | 0.066989 | 0.873844 | -0.14193 | 0.120662 | 0.98942 | 0.867678 | 1.128243 |
| 36 | ebi-a-GCST90092980 | ebi-a-GCST90001993 | PDL-1 on CD14+ CD16- monocyte \|\| id:ebi-a-GCST90001993 | \|\| id:ebi-a-GCST90092980 | Inverse variance weighted | 173 | -0.11676 | 0.064972 | 0.072314 | -0.24411 | 0.010581 | 0.889796 | 0.783403 | 1.010638 |
| 37 | ebi-a-GCST90092980 | ebi-a-GCST90001994 | CX3CR1 on CD14- CD16- \|\| id:ebi-a-GCST90001994 | \|\| id:ebi-a-GCST90092980 | Inverse variance weighted | 173 | 0.060526 | 0.065221 | 0.353401 | -0.06731 | 0.18836 | 1.062395 | 0.934908 | 1.207268 |
| 38 | ebi-a-GCST90092980 | ebi-a-GCST90002012 | CX3CR1 on CD14- CD16+ monocyte \|\| id:ebi-a-GCST90002012 | \|\| id:ebi-a-GCST90092980 | Inverse variance weighted | 173 | -0.0517 | 0.068642 | 0.451332 | -0.18624 | 0.082837 | 0.949613 | 0.830076 | 1.086365 |
| 39 | ebi-a-GCST90092980 | ebi-a-GCST90002017 | CCR2 on monocyte \|\| id:ebi-a-GCST90002017 | \|\| id:ebi-a-GCST90092980 | Inverse variance weighted | 173 | 0.026312 | 0.081307 | 0.74623 | -0.13305 | 0.185675 | 1.026661 | 0.875421 | 1.20403 |
| 40 | ebi-a-GCST90092980 | ebi-a-GCST90002024 | CD4 on naive CD4+ T cell \|\| id:ebi-a-GCST90002024 | \|\| id:ebi-a-GCST90092980 | Inverse variance weighted | 173 | 0.043103 | 0.073225 | 0.556101 | -0.10042 | 0.186623 | 1.044046 | 0.90446 | 1.205173 |
| 41 | ebi-a-GCST90092980 | ebi-a-GCST90002037 | CD80 on plasmacytoid Dendritic Cell \|\| id:ebi-a-GCST90002037 | \|\| id:ebi-a-GCST90092980 | Inverse variance weighted | 173 | 0.032759 | 0.078777 | 0.677521 | -0.12164 | 0.187161 | 1.033302 | 0.885464 | 1.205822 |
| 42 | ebi-a-GCST90092980 | ebi-a-GCST90002043 | CD45 on CD33+ HLA DR+ CD14dim \|\| id:ebi-a-GCST90002043 | \|\| id:ebi-a-GCST90092980 | Inverse variance weighted | 173 | 0.091474 | 0.097251 | 0.346911 | -0.09914 | 0.282086 | 1.095788 | 0.905618 | 1.325893 |
| 43 | ebi-a-GCST90092980 | ebi-a-GCST90002056 | CD8 on Effector Memory CD8+ T cell \|\| id:ebi-a-GCST90002056 | \|\| id:ebi-a-GCST90092980 | Inverse variance weighted | 173 | 0.024127 | 0.077734 | 0.756273 | -0.12823 | 0.176485 | 1.02442 | 0.87965 | 1.193016 |
| 44 | ebi-a-GCST90092980 | ebi-a-GCST90002110 | HLA DR on CD33dim HLA DR+ CD11b+ \|\| id:ebi-a-GCST90002110 | \|\| id:ebi-a-GCST90092980 | Inverse variance weighted | 173 | 0.113229 | 0.107828 | 0.293678 | -0.09811 | 0.324572 | 1.119888 | 0.906546 | 1.383438 |
| 45 | ebi-a-GCST90092980 | ebi-a-GCST90002116 | HLA DR on B cell \|\| id:ebi-a-GCST90002116 | \|\| id:ebi-a-GCST90092980 | Inverse variance weighted | 173 | -0.13131 | 0.103181 | 0.203149 | -0.33355 | 0.070923 | 0.876945 | 0.71638 | 1.073499 |

**Table S9:** Reverse Mendelian randomization analysis of the causal relationship between fatty acid levels and immune cell traits.

|  | id.exposure | id.outcome | outcome | exposure | method | nsnp | b | se | pval | lo_ci | up_ci | or | or_lci95 | or_uci95 |
| --- | --- | --- | --- | --- | --- | --- | --- | --- | --- | --- | --- | --- | --- | --- |
| 1 | ebi-a-GCST90001473 | ebi-a-GCST90092987 | Total fatty acid levels \|\| id:ebi-a-GCST90092987 | \|\| id:ebi-a-GCST90001473 | Inverse variance weighted | 92 | 0.003233 | 0.00252 | 0.19963 | -0.00171 | 0.008172 | 1.003238 | 0.998294 | 1.008206 |

**Table S10:** Heterogeneity test for fatty acid levels and immune cell traits.

|  | id.exposure | id.outcome | outcome | exposure | method | Q | Q_df | Q_pval |
| --- | --- | --- | --- | --- | --- | --- | --- | --- |
| 1 | ebi-a-GCST90092980 | ebi-a-GCST90001473 | CD62L- CD86+ myeloid Dendritic Cell %Dendritic Cell \|\| id:ebi-a-GCST90001473 | \|\| id:ebi-a-GCST90092980 | MR Egger | 161.5878 | 166 | 0.582248 |
| 2 | ebi-a-GCST90092980 | ebi-a-GCST90001473 | CD62L- CD86+ myeloid Dendritic Cell %Dendritic Cell \|\| id:ebi-a-GCST90001473 | \|\| id:ebi-a-GCST90092980 | Inverse variance weighted | 161.7794 | 167 | 0.59952 |

**Table S11:** Horizontal pleiotropy test for fatty acid levels and immune cell traits.

|  | id.exposure | id.outcome | outcome | exposure | egger_intercept | se | pval |
| --- | --- | --- | --- | --- | --- | --- | --- |
| 1 | ebi-a-GCST90092980 | ebi-a-GCST90001473 | CD62L- CD86+ myeloid Dendritic Cell %Dendritic Cell \|\| id:ebi-a-GCST90001473 | \|\| id:ebi-a-GCST90092980 | 0.002212 | 0.005053 | 0.662153 |

**Table S12:** Multivariable Mendelian randomization analysis of the causal relationship between fatty acids, immune cells, and coronary atherosclerosis.

|  | id.exposure | exposure | id.outcome | outcome | nsnp | b | se | pval | lo_ci | up_ci | or | or_lci95 | or_uci95 |
| --- | --- | --- | --- | --- | --- | --- | --- | --- | --- | --- | --- | --- | --- |
| 1 | ebi-a-GCST90001473 | CD62L- CD86+ myeloid Dendritic Cell %Dendritic Cell \|\| id:ebi-a-GCST90001473 | finn-b-I9_CORATHER | Coronary atherosclerosis \|\| id:finn-b-I9_CORATHER | 35 | 0.021463 | 0.008293 | 0.00965 | 0.005209 | 0.037717 | 1.021695 | 1.005223 | 1.038437 |
| 2 | ebi-a-GCST90092987 | Total fatty acid levels \|\| id:ebi-a-GCST90092987 | finn-b-I9_CORATHER | Coronary atherosclerosis \|\| id:finn-b-I9_CORATHER | 177 | 0.277284 | 0.053385 | 2.06E-07 | 0.17265 | 0.381917 | 1.31954 | 1.18845 | 1.465091 |
