# Supplementary figures and images for: Causal Mediation of Immune Cells and Fatty Acids in Coronary Atherosclerosis: Insights From Mendelian Randomization Analysis
Source: Hum Mutat. 2026 May 23;2026:1253577. doi: 10.1155/humu/1253577 (PMC13197653; doi:10.1155/humu/1253577)

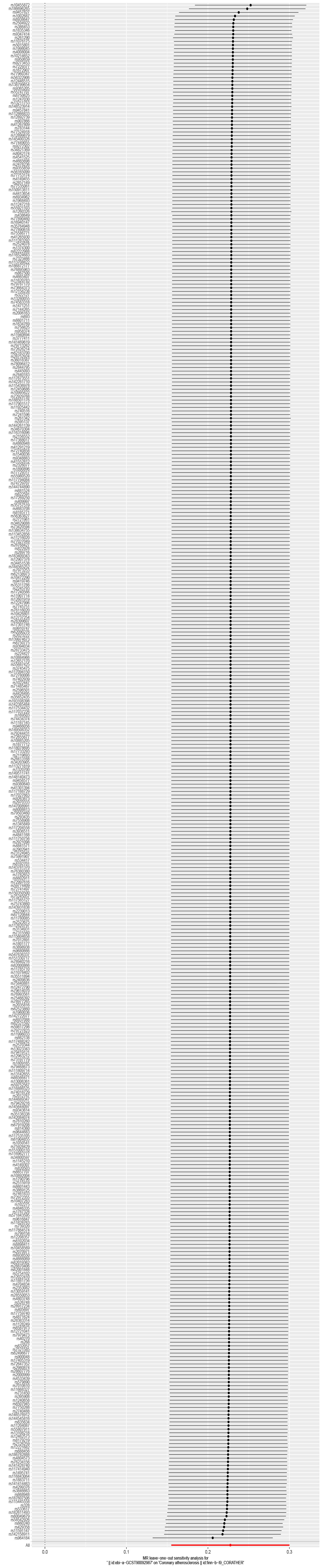

Supplement: Supplementary file 2 — Supporting Information 2 Figure S1: Leave‐one‐out (LOO) analyses. [file HUMU-2026-1253577-s002.tif]
